# Resistance evaluation of Chinese wild *Vitis* genotypes against *Botrytis cinerea* and different responses of resistant and susceptible hosts to the infection

**DOI:** 10.3389/fpls.2015.00854

**Published:** 2015-10-26

**Authors:** Ran Wan, Xiaoqing Hou, Xianhang Wang, Jingwu Qu, Stacy D. Singer, Yuejin Wang, Xiping Wang

**Affiliations:** ^1^State Key Laboratory of Crop Stress Biology in Arid Areas, College of Horticulture, Northwest A&F UniversityYangling, China; ^2^Key Laboratory of Horticultural Plant Biology and Germplasm Innovation in Northwest China, Ministry of Agriculture, Northwest A&F UniversityYangling, China; ^3^Department of Agricultural, Food and Nutritional Science, University of AlbertaEdmonton, AB, Canada

**Keywords:** antioxidative system, *Botrytis*-*Vitis* interactions, Chinese wild *Vitis*, ROS, resistance evaluation

## Abstract

The necrotrophic fungus *Botrytis cinerea* is a major threat to grapevine cultivation worldwide. A screen of 41 *Vitis* genotypes for leaf resistance to *B. cinerea* suggested species independent variation and revealed 18 resistant Chinese wild *Vitis* genotypes, while most investigated *V. vinifera*, or its hybrids, were susceptible. A particularly resistant Chinese wild *Vitis*, “Pingli-5” (*V.* sp. [Qinling grape]) and a very susceptible *V. vinifera* cultivar, “Red Globe” were selected for further study. Microscopic analysis demonstrated that *B. cinerea* growth was limited during early infection on “Pingli-5” before 24 h post-inoculation (hpi) but not on Red Globe. It was found that reactive oxygen species (ROS) and antioxidative system were associated with fungal growth. O${}_{2}^{-}$ accumulated similarly in *B. cinerea* 4 hpi on both *Vitis* genotypes. Lower levels of O${}_{2}^{-}$ (not H_2_O_2_) were detected 4 hpi and ROS (H_2_O_2_ and O${}_{2}^{-}$) accumulation from 8 hpi onwards was also lower in “Pingli-5” leaves than in “Red Globe” leaves. *B. cinerea* triggered sustained ROS production in “Red Globe” but not in “Pingli-5” with subsequent infection progresses. Red Globe displayed little change in antioxidative activities in response to *B. cinerea* infection, instead, antioxidative activities were highly and timely elevated in resistant “Pingli-5” which correlated with its minimal ROS increases and its high resistance. These findings not only enhance our understanding of the resistance of Chinese wild *Vitis* species to *B. cinerea*, but also lay the foundation for breeding *B. cinerea* resistant grapes in the future.

## Introduction

The necrotrophic fungal pathogen *Botrytis cinerea* causes gray mold disease in a broad range of plant species, including grape. Grape production, of great economic importance in China, relies almost exclusively on European grapevine varieties (lv, 2013); however, these are currently threatened by gray mold disease, especially with the rapid development of protected cultivation (Zhang, 2011; lv, 2013). Although agronomic, genetic, and biological approaches have been proposed to limit yield losses caused by gray mold, disease management is still largely based on chemical control (Angelini et al., 2014), which is not sustainable.

*B. cinerea* is one of the most comprehensively studied necrotrophic plant pathogens which can produce ROS and simultaneously induce host oxidative burst (van Kan, 2006). ROS, such as superoxide and hydrogen peroxide, can delay, or accelerate pathogen proliferation (Temme and Tudzynski, 2009; Afzal et al., 2014), and participate in cell wall modification, programmed cell death and the integration of many different signaling networks (Serrano et al., 2014). In addition, it has also been proposed that they may work as dynamic signaling molecules (Torres et al., 2006; Mittler et al., 2011). Thus, ROS play important and multifaceted roles during the interaction between *B. cinerea* and its plant hosts (Lamb and Dixon, 1997; De Tullio, 2010).

There is considerable evidence that *B. cinerea* can overturn the ROS stress induced in planta to assist its invasion of plant tissues (Govrin and Levine, 2000; Temme and Tudzynski, 2009). ROS have been reported to reduce resistance and accelerate expansion of disease lesions during *B. cinerea*-*Nicotiana benthamiana* interactions (Asai and Yoshioka, 2009). Tomato (*Solanum lycopersicum*) plants overexpressing the transcription factor *SlSHINE3*, which regulates cuticle production, were observed to be more resistant to *B. cinerea* with lower levels of ROS production and more cuticles than wild-type plants (Buxdorf et al., 2014). Nevertheless, the roles of ROS in the interaction between *B. cinerea* and its hosts remain controversial. For example, an induction of oxidative burst resulted in enhanced resistance against *B. cinerea* in *A. thaliana* with the application of the herbicide paraquat (Tierens et al., 2002), and a timely hyperinduction of H_2_O_2_ in the *sitiens* tomato mutant (deficient in abscisic acid (ABA) synthesis) effectively blocked infection by the pathogen (Asselbergh et al., 2007). Moreover, *A. thaliana* ABA or wax biosynthesis mutants, accompanied by an increased cuticular permeability, were reported to produce ROS earlier and in higher amounts, also showing increased resistance (L'Haridon et al., 2011; Serrano et al., 2014). In another study using bean (*Glycine max*) leaves, it was shown that the secondary oxidative burst was much stronger following challenge by a non-aggressive *B. cinerea* strain than by an aggressive strain, indicating that ROS-mediated responses have the capacity to block infection by the pathogen (Urbanek et al., 1996).

Despite numerous studies those have been conducted regarding the role of ROS in plant-*B. cinerea* interactions, the importance of ROS generation during *B. cinerea* invasion of grapevine has not been extensively examined. The application of bacterial rhamnolipids or BcPG1 (an endopolygalacturonase from *B. cinerea*) to *V. vinifera* was reported to improve resistance to *B. cinerea* by inducing ROS production and the expression of genes involved in defense through different signal pathways (Vandelle et al., 2006; Varnier et al., 2009). Similarly, treatment of grape cells with oligogalacturonides (Aziz et al., 2004) or bacteria, such as *Pseudomonas fluorescens* and *Pantoea agglomerans*, or extracts from these bacteria (Verhagen et al., 2010, 2011), triggered an oxidative burst in tandem with improving resistance to *B. cinerea* to varying degrees. Moreover, Gabler et al. (2003) found that *V. rotundifolia* and *V. labrusca* were highly resistant, while cultivars of *V. vinifera* were highly susceptible to *B. cinerea*. However, little is known about the potential sources and mechanisms of resistance in grapevines to *B. cinerea*. China is one of the major centers of origin of *Vitis* species (Wang et al., 1995, 1998), and the rich Chinese wild *Vitis* germplasm has been largely utilized for grape breeding programs due to its many desirable characteristics, such as resistance to a variety of fungal diseases and its ability to be easily crossed with *V. vinifera* than the multi-disease resistant *Muscadinia rotundifolia* (Luo and He, 2004).

In this study, *B. cinerea* resistance levels of Chinese wild *Vitis* are reported and the time course of colonization by *B. cinerea* on the leaves of highly resistant and susceptible *Vitis* genotypes is described. Histochemical and physiological evidence for the role of ROS and antioxidative systems in *Vitis*-*B. cinerea* interactions is presented. Taken together, our data provide a foundation for elucidating the events leading to resistance of Chinese wild *Vitis* to *B. cinerea* and for the future breeding of grape genotypes resistant to this pathogen.

## Materials and methods

### Plant and fungal material

Eleven Chinese wild *Vitis* species and four other *Vitis* species, totaling 41 genotypes, including 30 Chinese wild *Vitis* species, seven *V. vinifera* species, as well as *V. riparia Michanwx*. “*Hean-3,”* two *V. vinifera* × *V. labrusca* cv. “Kyoho” and “NO. 8 Hutai” and *V. vinifera* × *V. amurensis* cv. “Beichun,” were evaluated from 2011 to 2013 (Table 1). The germplasm was maintained in the vineyard overseen by the grape germplasm and breeding program of Northwest A&F University, Shaanxi, China.

**Table 1:** **Laboratory evaluation results (including macroscopic and light microscopic examination) of 41 *Vitis* genotypes against *Botrytis Cinerea* from 2011 to 2013**.

**Species**	**Names of genotypes**	**Disease Severity^c^**	**Scores^d^**	**Rank of scores**	**Resistance levels^e^**	**Rates of germination (%)**	**Rates of infection (%)**	**Macroscopic mycelium**	**New sporation**
*V. amurensis* Rupr	Huaxian-11	5.91±1.86	1.56	35	R	2.91	0.67	–^f^	–
*V. amurensis* Rupr	Taishan-11	4.58±0.79	1.44	36	HR	12.07	3.45	–	–
*V. amurensis* Rupr	Zuoshan-1	38.99±1.31	3.78	20	S	63.05	28.14	√^g^	–
*V. amurensis* Rupr	Tonghua-3	0.20±0.05	1.00	40	HR	13.10	6.35	–	–
*V. amurensis* Rupr	Shuangyou^b^	0.18±0.05	1.00	41	HR	13.17	7.32	–	–
*V. romanetii* Roman.	Pingli-2	46.98±1.20	4.89	11	S	63.71	47.18	√	√
*V. romanetii* Roman.	Baihe-22	29.71±2.73	3.44	21	S	56.14	38.16	–	–
*V. romanetii* Roman.	Liuba-11	46.59±2.09	4.78	13	S	46.67	42.22	√	–
*V. romanetii* Roman.	Jiangxi-2	49.33±3.36	4.89	12	S	61.90	52.86	√	√
*V. quinquangularis* Rehd.	Shang-24	70.48±5.81	6.00	3	HS	51.85	43.70	√	–
*V. quinquangularis* Rehd.	Taishan-12	21.96±2.18	3.00	25	R	19.38	9.69	–	–
*V. quinquangularis* Rehd.	83-4-85^a^	21.38±2.95	2.89	27	R	12.39	7.34	√	–
*V. quinquangularis* Rehd.	83-4-96^a^	42.9±2.73	4.33	17	S	42.35	14.12	–	–
*V piasezkii* Maxim	Liuba-6	18.04±0.59	3.00	24	R	29.74	21.24	–	–
*V piasezkii* Maxim	Liuba-7	16.68±1.19	2.78	29	R	21.91	16.36	–	–
*V piasezkii* Maxim	Gansu-91	12.64±0.66	2.11	33	R	30.26	20.61	–	–
*V. adstricta* Hance	Taishan-1	15.14±1.14	2.56	31	R	23.69	16.47	–	–
*V. adstricta* Hance	Taishan-2	2.08±0.43	1.00	38	HR	23.49	15.36	–	–
*V. adstricta* Hance	Anlin-3	16.74±1.65	2.78	30	R	46.88	38.92	√	–
*V. davidii* Foex	Lueyang-4	55.63±2.60	5.11	10	S	64.58	55.56	√	√
*V. davidii* Foex	Ningqiang-6	59.79±1.10	5.56	7	HS	77.03	70.27	√	√
*V. davidii* Foex	Tangwei^b^	7.00±1.52	1.89	34	R	74.65	62.50	√	–
*V. davidii* Foex	Fujian-4	46.32±3.09	4.56	15	S	54.01	36.36	√	–
*V. pseudoreticulata* W.T. Wang	Guangxi-1	22.97±2.57	3.11	22	R	26.06	15.49	–	–
*V. pseudoreticulata* W.T. Wang	Hunan-1	61.40±3.97	5.67	5	HS	82.95	60.08	√	√
*V. sp.* (Maihuang grape)	Baihe-41	28.04±0.86	3.00	26	R	38.10	20.95	–	–
*V. sp.* (Maihuang grape)	Baihe-36-2	16.54±1.37	2.89	28	R	37.67	22.26	√	–
*V. davidii* var. cyanocarpa Sarg.	Zhenan-3	40.43±2.12	4.00	19	S	43.27	33.82	√	–
*V. sp.* (Qinling grape)	Pingli-5	3.70±0.90	1.22	37	HR	28.06	12.23	–	–
*V. yenshanensis*	Yanshan-1	0.36±0.16	1.00	38	HR	29.19	9.81	–	–
*V. vinifera* L.	NO19 Xinong	38.27±2.35	4.00	18	S	58.70	51.09	√	√
*V. vinifera* L.	Rizamat	24.14±2.62	3.00	23	R	25.58	21.14	√	–
*V. vinifera* L.	Hongmu Nage	46.63±3.46	4.67	14	S	87.50	82.95	√	√
*V. vinifera* L.	Zao Jinxiang	13.06±0.89	2.11	32	R	46.54	37.11	–	–
*V. vinifera* L.	Muscat Hamburg	59.69±6.12	5.44	9	S	86.08	64.64	√	√
*V. vinifera* L.	Red Face Seedless	60.59±2.17	5.56	6	HS	72.40	61.99	√	√
*V. vinifera* L.	Red Globe	72.25±3.57	6.11	2	HS	88.77	70.01	√	√
*V. riparia Michawx*	Hean-3	43.57±2.13	4.33	16	S	84.85	55.56	√	√
*V. vinifera* L. × *V.* labrusca L.	Kyoto	58.11±6.49	5.56	8	HS	72.88	54.95	√	√
*V. vinifera* L. × *V.* labrusca L.	NO8 Hutai	77.82±6.17	6.33	1	HS	79.54	70.96	√	√
*V. vinifera* L. × *V.* amurensis Rupr	Beichun	66.90±6.17	5.89	4	HS	65.26	54.21	√	√

a The genotypes were selected from seedlings of V. qinquangularis (Wang et al., 1995).

b The flower type of the genotypes were hermaphrodites under natural conditions (Wang et al., 1995).

c Disease Severity: the average percentage of spreading lesions determined by observing at least 10 leaves in each repeated experiment from 2011 to 2013.

d Score: disease severity was scored as previously described (Liu et al., 2003; Patykowski, 2006; Foyer and Noctor, 2013).

e Resistance level: Highly Resistant (HR: scores of 0–1.50); Resistant (R: scores of 1.51–3.50); Susceptible (S: scores of 3.51–5.50); Highly Susceptible (HS: scores of 5.51–7).

f √Mycelium or sporulation were observed by the naked eye on leaf surfaces.

g –No mycelium or sporulation was observed by the naked eye on leaf surfaces.

*B. cinerea* was isolated from “Red Globe” (*V. vinifera*) in the greenhouse and was maintained on Potato Glucose Agar medium in the dark at 22°C. After 21 days, conidia were washed down with distilled water, counted, and added to the inoculation solution at concentrations detailed in the following sections. Conidia were pre-germinated for 2 h at 22°C before inoculations were performed (Asselbergh et al., 2007).

### Detached leaf evaluation, fungal colonization experiments, and ROS measurements

Detached leaf assays were carried out using leaves of a similar age and size (leaves at nodes 3 and 4, counted from the top) selected randomly from vines. Detached leaves were washed carefully, first under tap water and then distilled water, and were then quickly transferred to a bed of 0.8% agar in trays and then uniformly sprayed with *B. cinerea* conidia suspension. Trays were covered with preservative film to ensure a relative humidity of 90–100%. All leaves from control (sprayed with distilled water) and inoculation treatments were incubated in the dark for the first 24 h and then in a light/dark (12/12 h) regime at 22°C (Audenaert et al., 2002; Windram et al., 2012).

To evaluate detached leaves (laboratory evaluation), at least 18 leaves from three biological replicates of each genotype were tested. Four days after inoculation, the infection was evaluated by counting the percentage of spreading lesions on each leaf. Before evaluation, the optimal inoculation solution and conidia concentration of *B. cinerea* were determined. The conidia germination in solutions with different glucose (Glc) and phosphate concentrations was determined under a light microscope after 6 and 24 h. The four solutions tested in this study were: (i) sterile; (ii) 1 × 10^6^ spores mL^−1^, 0.1 M Glc, 67 mM KH_2_PO_4_; (iii) 1 × 10^6^ spores mL^−1^, 0.05 M Glc, 33 mM KH_2_PO_4_; and (iv) 1 × 10^6^ spores mL^−1^, 0.01 M Glc, 6.7 mM KH_2_PO_4_ (Audenaert et al., 2002). Detached leaves of Red Globe and four Chinese wild grapevines, “Shang-24” (*V. quinquangularis*), “Hunan-1” (*V. pseudoreticulata*), “Taishan-2” (*V. adstrica*), “Baihe-41” (*V.* sp. [Maihuang grape]) were evaluated after infection with conidia suspensions of different concentrations (1 × 10^7^ spores mL^−1^; 1.5 × 10^6^ spores mL^−1^; 5 × 10^5^ spores mL^−1^ and 5 × 10^4^ spores mL^−1^).

For time series experiments, single inoculated, and control leaves were sampled 4, 8, 12, 18, 24, 36, 48, 72, and 96 hpi (hours post-inoculation) in a randomized manner from each of three biological replicates, except in the case of samples used for DAB (diaminobenzidine) staining.

### Rating of disease severity

Disease severity was evaluated from 2011 to 2013 and scored as previously described (Liu et al., 2003; Poolsawat et al., 2012). Disease resistance levels of the different genotypes were classified as: Highly Resistant (HR: scores of 0–1.50); Resistant (R: scores of 1.51–3.50); Susceptible (S: scores of 3.51–5.50); or Highly Susceptible (HS: scores of 5.51–7.0).

### Light microscopy and scanning electron microscopy

To characterize the colonization of “Pingli-5” (HR, Highly Resistant) and “Red Globe” (HS, Highly Susceptible) by *B. cinerea*, 2–3 cm^2^ leaf pieces were collected at 4, 8, 12, 18, 24, 36, 48, 72, and 96 hpi, fixed, and decolorized in ethanol/trichloromethane (3:1, v/v) containing 0.15% (w/v) trichloroacetic acid, before clearing in saturated chloral hydrate, and were then stored in 20% glycerol. Samples were subsequently stained with aniline blue solution (for staining fungal tissues a blue color) and examined with an Olympus BX-51 microscope (Olympus Corporation, Japan). For each sample, fungal germination, and infection percentages were examined. For scanning electron microscopy (SEM), leaf tissues were cut into small pieces (0.5–1 cm^2^), fixed in 4% (v/v) glutaraldehyde in phosphate buffer (0.1 M, pH 6.8) for 12 h at 4°C, and rinsed in the same buffer four times for 10–15 min. After dehydration in a graded ethanol series (30, 50, 70, 80, 90, 100%, v/v), the samples were then critical-point dried, coated with gold in a sputter coater, and examined with a JEOL FESEM S-4800 scanning electron microscope at 15 kV (Cheng et al., 2012).

### Histochemical analysis of ROS responses

H_2_O_2_ and O${}_{2}^{-}$ were respectively detected by DAB and NBT (nitro blue tetrazolium) staining protocols, as previously described (ThordalChristensen et al., 1997; Wang et al., 2007) to compare the ROS responses of the two genotypes defined as HR and HS. Two to three centimeter^2^ leaf segments were immersed under direct light in a DAB solution (1 mg/mL with HCl acidifying to pH 3.8) 8 h before sample collection except for that samples 4 hpi were directly immersed in DAB solution once inoculated. The leaves were prepared for observation as described above. Leaf segments of the same size were collected directly into 0.1% (w/v) NBT solution in 10 mM phosphate buffer (pH 7.8) prior to a vacuum infiltration for 30 min and an exposure to direct light for 20 min. The NBT stained samples were then observed as above, except for the omission of aniline blue staining. The percentages of conidia, germ tubes, and infection sites exhibiting O${}_{2}^{-}$ or H_2_O_2_ accumulation were evaluated.

### Antioxidant enzyme extraction and activity assays

Crude protein extracts to assess superoxide dismutase (SOD) (Mittler et al., 2011) and peroxidase (POD) (Atkinson and Urwin, 2012) activities were isolated from approximately 0.5 g leaves using protocols described by Giannopolitis and Ries (1977). For SOD activity, briefly, 3.4 mL reaction mixtures comprising 50 mM sodium phosphate buffer (pH 7.0), 13 mM methionine, 75 μM NBT, 2 μM riboflavin, 0.1 mM EDTA, and 100 μl crude protein extract were illuminated for 20 min at 4000 Lux and then measured at 560 nm. POD activity was assayed as previously described (Maehly and Chance, 1954). Six-hundred Microliter crude protein extract added to a 3 mL reaction mixture comprising 0.05 M guaiacol and 2% H_2_O_2_ was measured at 470 nm.

Crude protein extracts for measuring catalase (CAT) (Atkinson and Urwin, 2012) activity were obtained from approximately 2.5 g leaves that was ground in 25 mL cold 0.2 M PBS buffer (pH 7.8). CAT activity was determined by measuring the consumption of H_2_O_2_ by KMnO_4_. The mixture of 3 mL crude protein extract, 2.5 mL 10% H_2_SO_4_ and 2.5 mL 0.1 M H_2_O_2_ were incubated for 10 min at 30°C and then titrated with 0.1 M KMnO_4_. Samples with 3 mL boiled extract in the reaction mixtures were used as controls. The consumption of 1.7 mL 0.1 M KMnO_4_ was assumed to be equal to 1.7 mg H_2_O_2_. The KMnO_4_ solution of 0.1 M was critically determined by 0.1 M oxalic acid GR (Maehly and Chance, 1954).

### Statistical analyses

All experiments were performed using three biological replicates. At least 300 conidia from eight to ten leaf sections per time point were examined in histopathological and histochemical sections. Means and standard errors were calculated from three independent experiments by Microsoft Excel (Microsoft Corporation) and significant differences and Duncan LSD analysis by a completely random design and correlation analyses of resistance evaluation data from 2011 to 2013 were performed using SPSS Statistics (Gabler et al., 2003; Poolsawat et al., 2012). All pictures were combined by Adobe Photoshop (Adobe Systems Incorporated).

## Results

### The optimum inoculum and concentration of *B. cinerea*

Since some *B. cinerea* isolates germinate readily in distilled water, while others require sugars to initiate an infection (Schumacher and Tudzynski, 2012), a comparative assay was performed to determine the optimal inoculation solution, as well as a moderate concentration of *B. cinerea* conidia to be used in the subsequent experiments (Figure S1). *V. vinifera* cv. “Red Globe” and *V. adstrica* “Taishan-2” have previously been tested and found to be HS and HR species, respectively. Three other genotypes “Shang-24” (*V. quinquangularis*), “Hunan-1” (*V. pseudoreticulata*) and “Baihe-41” (*V.* sp. [Maihuang grape]), were also randomly selected for the comparative assay with different concentrations of spores in sterile water (Figure S1A). The *B. cinerea* used in the present study performed substantially better for higher spore germination rate after 24 h in sterile water than in solutions of different Glc and KH_2_PO_4_ concentrations (Figure S1B). Inoculation of “Red Globe,” “Shang-24,” “Hunan-1” leaves with a 1 × 10^7^ mL^−1^ spore suspension all caused brownish spreading lesions that almost colonized the whole leaf area. When 5 × 10^4^ spores mL^−1^ was used, no spreading lesions were observed on “Baihe-41” and “Taishan-2.” Assay conditions should result in a moderately aggressive infection to distinguish different levels of resistance. Thus, 1 × 10^7^ spores mL^−1^ was evidently too aggressive, while 5 × 10^4^ spores mL^−1^ was too mild. Therefore, an inoculation with 1.5 × 10^6^ spores mL^−1^ in sterile water was opted for the subsequent analyses, which allowed us to detect both increases and decreases in disease severity, for its larger range of the percentages of spreading lesions on the different genotypes than 5 × 10^5^ spores mL^−1^.

### Chinese wild *Vitis* species exhibit different levels of resistance to *B. cinerea*

It has been established that the detached leaf assay in the laboratory gives similar results to field evaluations and that it is a reliable method for screening resistance of grapevine cultivars/lines and their hybrids (Wang et al., 1995; Liu et al., 2003; Poolsawat et al., 2012). According to our laboratory resistance evaluation of *B. cinerea*, whereby spreading leaf lesions (disease severities) were counted 4 days post-inoculation, Chinese wild *Vitis* species generally exhibited a greater degree of variation in their resistance to *B. cinerea* than other species did (Table 1). The data showed similarity in repeated tests and average disease severities varied significantly (*P* ≤ 0.05) among the different genotypes through completely random Duncan LSD analysis (Table 2), but no significant difference (P>0.05) was observed between years (2011 and 2013) using correlation analyses (Table S1).

**Table 2:** **Means ± standard deviations of 3 years of lesions percent ages on the leaves of 41 *Vitis* genotypes infected with *B. cinerea* over 3 years, along with significance analysis of disease severities**.

**Species**	**Names of genotypes**	**Means ± Deviation of lesion %**			*P* < 0.05^*^	*P* < 0.01 ^**^
		**2011**	**2012**	**2013**		
*V. amurensis* Rupr	Huaxian-11	6.11 ± 1.21	4.33 ± 2.44	7.29 ± 1.93	o	QR
*V. amurensis* Rupr	Taishan-11	4.49 ± 1.00	4.71 ± 0.52	4.53 ± 0.85	o	RS
*V. amurensis* Rupr	Zuoshan-1	39.7 ± 0.89	38.8 ± 1.12	38.47 ± 1.92	j	KL
*V. amurensis* Rupr	Tonghua-3	0.18 ± 0.06	0.18 ± 0.09	0.23 ± 0.13	p	S
*V. amurensis* Rupr	Shuangyou	0.20 ± 0.04	0.12 ± 0.03	0.21 ± 0.07	p	S
*V. romanetii* Roman.	Pingli-2	45.44 ± 0.85	47.89 ± 1.39	47.61 ± 1.35	fg	GH
*V. romanetii* Roman.	Baihe-22	30.30 ± 2.38	28.38 ± 3.89	30.45 ± 1.91	k	LM
*V. romanetii* Roman.	Liuba-11	45.56 ± 3.20	46.54 ± 1.77	47.66 ± 1.29	fg	GHI
*V. romanetii* Roman.	Jiangxi-2	50.93 ± 1.04	50.35 ± 2.57	46.71 ± 6.48	fg	GH
*V. quinquangularis* Rehd.	Shang-24	68.27 ± 2.67	66.93 ± 8.13	76.23 ± 6.62	abc	ABC
*V. quinquangularis* Rehd.	Taishan-12	20.81 ± 1.58	20.38 ± 2.34	24.69 ± 2.63	l	MNO
*V. quinquangularis* Rehd.	83-4-85	21.52 ± 1.72	23.50 ± 2.61	19.12 ± 4.52	lm	NO
*V. quinquangularis* Rehd.	83-4-96	43.44 ± 2.02	43.80 ± 2.87	41.46 ± 3.30	hi	IJ
*V piasezkii* Maxim	Liuba-6	18.53 ± 0.55	17.28 ± 0.62	18.38 ± 0.59	l	MNO
*V piasezkii* Maxim	Liuba-7	16.12 ± 0.29	17.14 ± 2.10	16.79 ± 1.19	lm	NO
*V piasezkii* Maxim	Gansu-91	14.13 ± 0.81	12.38 ± 0.68	11.42 ± 0.49	n	P
*V. adstricta* Hance	Taishan-1	14.30 ± 1.30	15.27 ± 1.69	15.84 ± 0.44	m	O
*V. adstricta* Hance	Taishan-2	1.87 ± 0.17	2.23 ± 0.51	2.13 ± 0.62	p	S
*V. adstricta* Hance	Anlin-3	16.39 ± 1.08	17.65 ± 1.61	16.18 ± 2.25	lm	NO
*V. davidii* Foex	Lueyang-4	56.35 ± 1.59	53.98 ± 3.08	56.57 ± 3.12	f	EFG
*V. davidii* Foex	Ningqiang-6	60.65 ± 0.54	59.38 ± 1.92	59.34 ± 0.83	de	CDE
*V. davidii* Foex	Tangwei	6.38 ± 0.57	5.70 ± 2.33	8.92 ± 1.65	n	PQ
*V. davidii* Foex	Fujian-4	45.88 ± 2.95	47.54 ± 5.31	45.53 ± 1.02	gh	HI
*V. pseudoreticulata* W.T. Wang	Guangxi-1	21.78 ± 2.34	20.75 ± 1.82	26.39 ± 3.54	l	MN
	Hunan-1	61.50 ± 2.46	60.12 ± 4.25	62.59 ± 5.20	cde	BCD
*V. sp.* (Maihuang grape)	Baihe-41	26.68 ± 1.72	28.37 ± 0.36	29.06 ± 0.49	l	MNO
*V. sp.* (Maihuang grape)	Baihe-36-2	17.16 ± 1.58	16.79 ± 1.80	15.68 ± 0.72	lm	NO
*V. davidii* var. cyanocarpa Sarg.	Zhenan-3	40.68 ± 2.13	38.95 ± 2.80	41.67 ± 1.42	ij	JK
*V. sp.* (Qinling grape)	Pingli-5	3.91 ± 1.12	3.57 ± 0.62	3.61 ± 0.95	op	RS
*V. yenshanensis*	Yanshan-1	0.26 ± 0.14	0.34 ± 0.23	0.48 ± 0.11	p	S
*V. vinifera* L.	NO. 19 Xinong	39.91 ± 2.91	40.53 ± 1.71	34.37 ± 2.44	ij	JK
*V. vinifera* L.	Rizamat	25.04 ± 1.78	24.40 ± 3.40	22.97 ± 2.69	l	MNO
*V. vinifera* L.	Hongmu Nage	45.68 ± 0.74	44.93 ± 5.36	49.27 ± 4.28	gh	GHI
*V. vinifera* L.	Zao Jinxiang	12.70 ± 0.33	13.00 ± 1.60	13.48 ± 0.74	n	P
*V. vinifera* L.	Muscat Hamburg	61.55 ± 3.75	63.30 ± 5.60	54.22 ± 9.02		e
*V. vinifera* L.	Red Face Seedless	61.10 ± 3.48	58.97 ± 1.13	61.70 ± 1.89	de	CDE
*V. vinifera* L.	Red Globe	71.90 ± 1.26	69.37 ± 2.91	75.47 ± 6.55	ab	AB
*V. riparia Michawx*	Hean-3	47.85 ± 2.33	43.95 ± 3.13	38.90 ± 0.92	hi	IJ
*V. vinifera* L. × *V.* labrusca L.	NO. 8 Hutai	69.98 ± 6.78	76.33 ± 7.82	87.15 ± 3.90	a	A
*V. vinifera* L. × *V. labrusca* L.	Kyoho	61.33 ± 6.22	55.53 ± 5.13	57.47 ± 8.12	de	CDE
*V. vinifera L. × V. amurensis Rupr*	Beichun	65.39 ± 3.54	67.45 ± 13.36	67.86 ± 1.62	bcd	ABCD

*, ** Significance at P ≤ 0.05 or P ≤ 0.01, respectively. Different letters associated with each level of disease severity indicates significant differences at P ≤ 0.05 or P ≤ 0.01.

Among the 20 genotypes that were classified as resistant at least (scores between 0 and 3.50), 18 were Chinese wild *Vitis* genotypes, which was approximately 70% of all 41 genotypes tested. The remaining 21 were susceptible genotypes at least (scores between 3.51 and 7.0) in which only 10 belonged to Chinese wild *Vitis* species (Table 1). The disease severity of the three most highly resistant genotypes (HR, scores between 0.00 and 1.50) was less than 0.5%, and infection lesions were rarely to be observed (Table 1). In contrast, leaves of the most susceptible genotypes (HS, scores between 5.51 and 7.0) showed soft-rot and new sporulation (Table 1).

**Figure 1:**
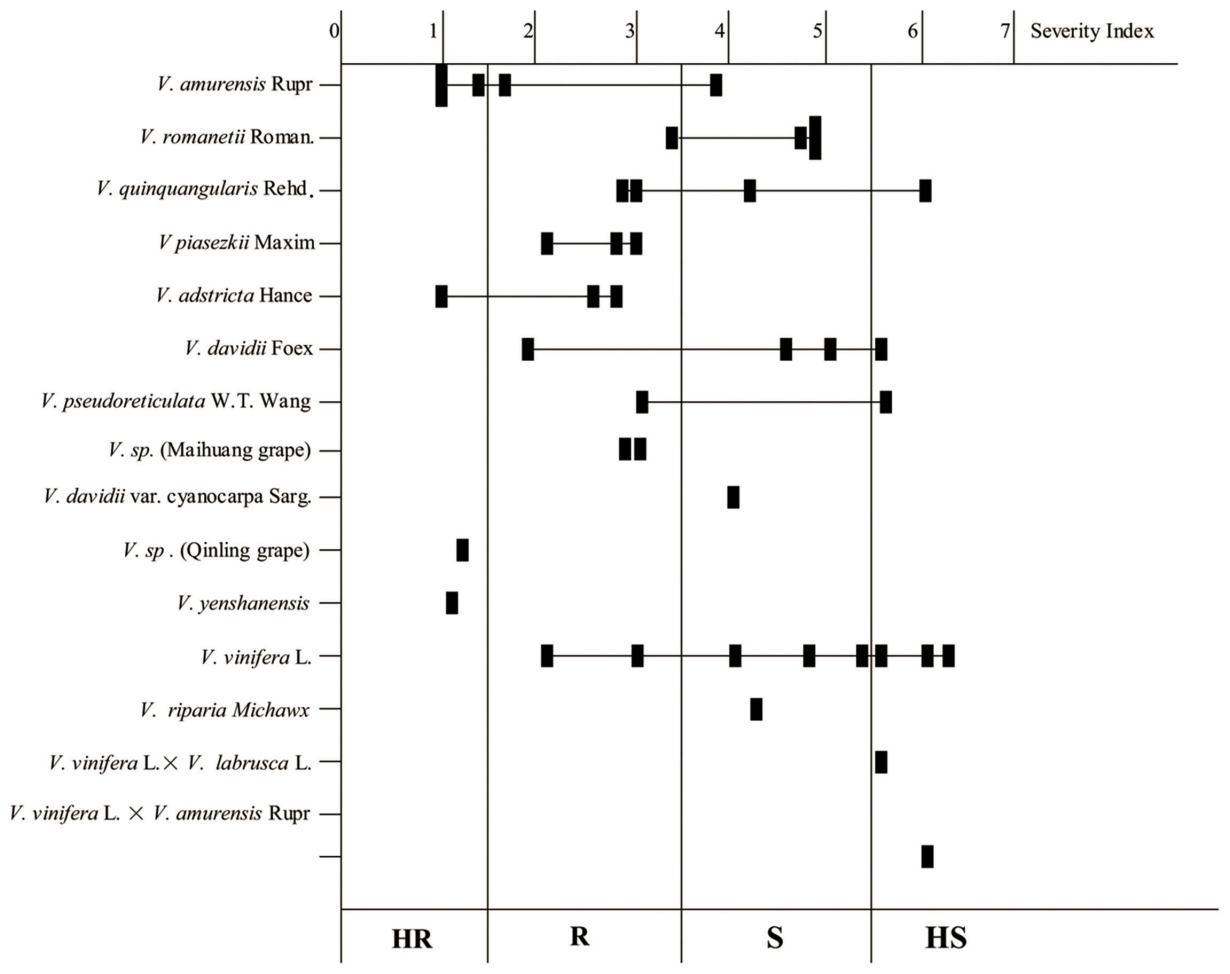
**Histogram showing the resistance levels of the 41 tested *Vitis* genotypes to *B. cinerea***. Solid squares represent the average of the severity index. HR, Highly Resistant (Scores: 0–1.5); R, Moderately Resistant (Scores: 1.51–3.5); S, Moderately Susceptible (Scores: 3.51–5.5); HS, Highly Susceptible to (Scores: 5.51–7.0).

Variation in the resistance levels of Chinese wild *Vitis* to *B. cinerea* is shown in Figure 1, indicating that resistance diversity is reasonably species independent at least to an extent. Little or no resistance was observed in the widely grown *V. vinifera* cultivars. Indeed, five of the eight HS genotypes were cultivars of *V. vinifera* or its hybrids. Four *V. romanetii* genotypes and three *V. davidii* genotypes but “Tangwei” were classified as susceptible at least, while four of five *V. amurensis* genotypes were resistant at least. Furthermore, all three *V. piasezkii*, three *V. adstricta* and two *V.* sp. genotypes were classified as R (scores between 1.51 and 3.5) or HR, as were *V.* sp. (Qinling grape) and *V. yenshanensis*, although there was only one representative. It is noteworthy that all six genotypes identified as HR were Chinese wild *Vitis*: “Pingli-5” (*V.* sp. [Qinling grape]); “Yanshan-1” (*V. yenshanensis*); “Taishan-2” (*V. adstricta*); and three *V. amurensis* genotypes (“Shuangyou,” “Tonghua-3” and “Taishan-11”).

All the susceptible genotypes of *V. vinifera* or Chinese wild *Vitis* showed macroscopic mycelium 4 days after infection (Table 1). However, there were also five genotypes classified as R that showed minimal formation of mycelia, and the spreading lesions were far smaller than those seen in the susceptible genotypes. The fungus underwent new sporulation on 14 genotypes, half of which was classified as S (scores between 3.51 and 5.5) and the other half as HS, and neither mycelia nor sporulation were observed on leaves of any HR genotype. Germination and infection rates of all 41 evaluated genotypes were also measured, with germination rates referring to the percentages of germinated conidia of total counted conidia, and infection rates indicating the percentages of successful infection of total counted germinated conidia (Table 1). Most germination and infection rates on HR leaves were less than 20%, while those on R leaves were typically 15–50% and 20–40%, respectively. The rates with S genotypes were at least 50 and 20–60%, respectively, while on HS plants they were more than 60 and 50–80%, respectively. However, there were some conflicting observations: for example, although the germination rate on leaves of the susceptible “83-4-96” (*V. quinquangularis*) was 42.4% and spreading lesions reached 42.9%, the infection rate was only 14.1% that was even lower than the R genotype “Gansu-91” (*V. piasezkii*) (Table 1). Since disease development is not only related to infection rates but also to post-penetration processes (Elad, 1997), the latter genotype was suggested being more sensitive to *B. cinerea* because lower infection rates caused more lesions. Despite of that, the data from the different analyses were generally corroborated with each other, so the laboratory analysis combining with the macroscopic and microscopic evaluation should give important insights into the resistance levels of the tested genotypes.

**Figure 2:**
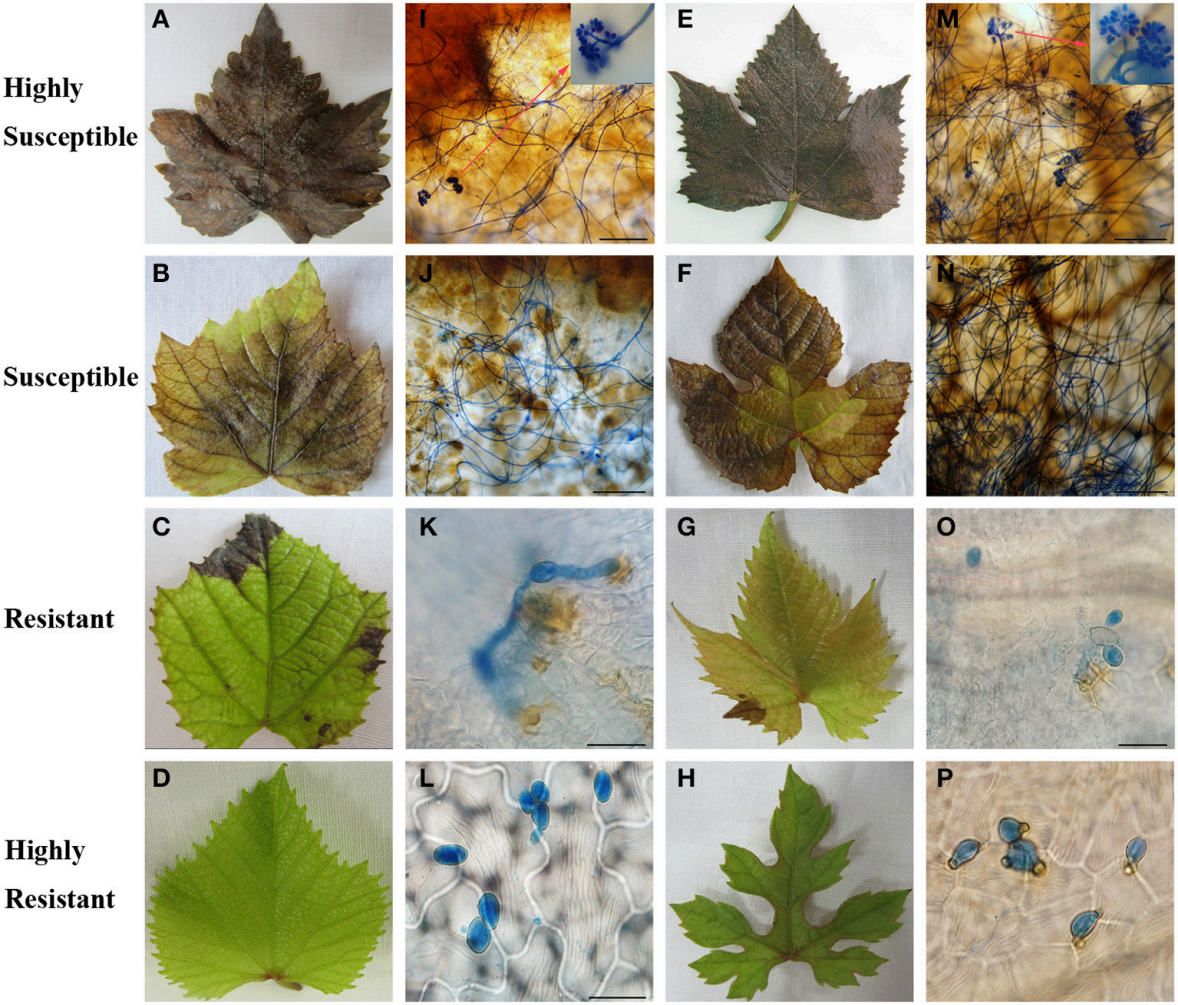
**Macroscopic (A–H) and microscopic (I–P) evaluation of two representative *Vitis* genotypes from each level of *B. cinerea* resistance, respectively**. Highly susceptible “Red Globe” (*V. Vinifera*) and “Beichun” (*V. Vinifera* × *V. amurensis* Rupr) are shown in **(A,I)** and **(E,M)**, respectively. Red arrows in **(I,M)** show new sporulation events at sites indicated. Susceptible *V. davidii* var. “Zhenan-3” and *V. romanetii* “Pingli-2” are shown in **(B,J)** and **(F,N)**, respectively. *V. quinquangularis* “83-4-85” and *V piasezkii* “Gansu-91” represent resistant genotypes and are shown in **(C,K)** and **(G,O)**, respectively. *V. amurensis* “Tonghua-3” and *V. sp.* (Qinling grape) “Pingli-5” are highly resistant and are shown in **(D,L)** and **(H,P)**, respectively. Scale bars: **(I,J,M,N)**: 50 μm; **(K,L,O,P)**: 20 μm. One representative leaf of three biological replicates is shown for each time point. Samples were collected 4 days after inoculation.

Two representative genotypes from the HR, R, S, and HS classes were selected to further compare the macroscopic and microscopic growth of *B. cinerea* 4 days after inoculation (Figure 2). The leaves of “Red Globe” (Figures 2A,B) and “Beichun” (Figures 2E,M), two HS genotypes, had entirely decayed and were covered with mycelium, and new conidia with infection rates were 70 and 50%, respectively (Table 1). The S genotypes, *V. davidii* var. “Zhenan-3” (Figures 2B,J) and *V. romanetii* “Pingli-2” (Figures 2F,N) had numerous spreading lesions with mycelia and few new conidia, and with infection rates of 34 and 47%, respectively, and spreading lesions of 47 and 40%, respectively (Table 1). The R genotypes *V. quinquangularis* “83-4-85” (Figures 2C,K) and *V. piasezkii* Gansu-91 (Figures 2G,O) produced considerably fewer limited necrotic lesions than the S and HS genotypes. The conidia on their leaves were observed to penetrate with rates of 7 and 21%, respectively (Table 1); however, the secondary hyphae either did not develop or were very short, indicating restricted *B. cinerea* proliferation. Finally, leaves of the HR genotypes, *V. amurensis* “Tonghua-3” (Figures 2D,L) and *V. sp.* (Qinling grape) “Pingli-5” (Figures 2H,P), had few lesions with the percentages of 0.2 and 4%, respectively. Germination rates of 13 and 28% and infection rates of 6 and 12% were also extremely low (Table 1). Abnormal germ tubes (Figure 2P) that were extremely short as well as hollow or collapsed conidia were observed to varying degrees on the leaves of almost all the HR genotypes analyzed.

### *B. cinerea* growth on the HS “red globe” and the HR chinese wild *Vitis* “pingli-5”

**Figure 3:**
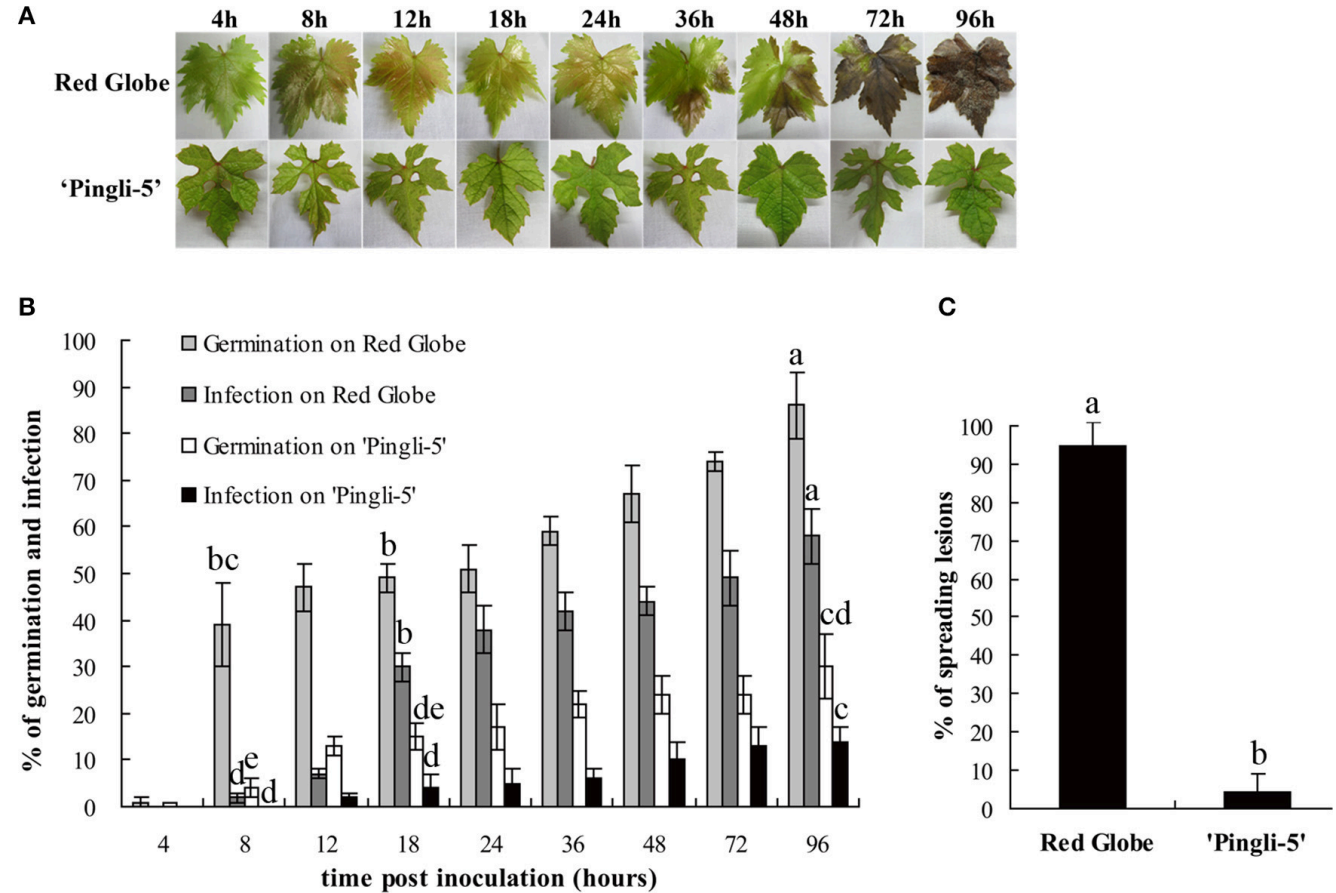
**Disease development after inoculation of leaves from the highly susceptible *V. Vinifera* “Red Globe” and the highly resistant Chinese wild *Vitis* “Pingli-5.” (A)** Pictures were taken 4, 8, 12, 18, 24, 36, 48, 72, and 96 h post-inoculation (hpi). Lesions were rarely observed on “Pingli-5,” while necrosis developed rapidly on “Red Globe” from 24 hpi onwards. One representative leaf of three replicates is shown for each time point. **(B)** Statistical analysis of conidia germination and infection rates. Lower rates of germination and invasion of *B. cinerea* were shown on “Pingli-5” than on “Red Globe.” At least 300 conidia were counted at each indicated time point. **(C)** Percentage of spreading lesions on “Red Globe” and “Pingli-5” leaves 4 days post-inoculation. Data represent the means of three experiments. Error bars denote standard deviations. Comparisons of statistical significance were made for the three indices. Different small alphabetical letters indicate statistically significant differences between different interactions of “Red Globe” and “Pingli-5” with *B. cinerea* at the indicated time points (Duncan's multiple range test; *P* < 0.05).

In this study, one of the most resistant Chinese wild *Vitis* genotypes, “Pingli-5,” and one of the most susceptible *V. vinifera*, “Red Globe,” were selected to characterize differences in their infection by *B. cinerea*. The first different visual symptoms were small, dark needle-like lesions 18 hpi on the upper leaf surface of “Red Globe” that were not present on “Pingli-5.” These subsequently developed into small necrotic lesions 24 hpi that expanded rapidly until 96 hpi, resulting in extensive tissue rot and new sporulation. Conversely, only a few necrotic spots were observed on “Pingli-5” leaves and these showed minimal expansion (Figure 3A), with about 5% necrosis compared to 95% on “Red Globe” leaves (Figure 3C).

**Figure 4:**
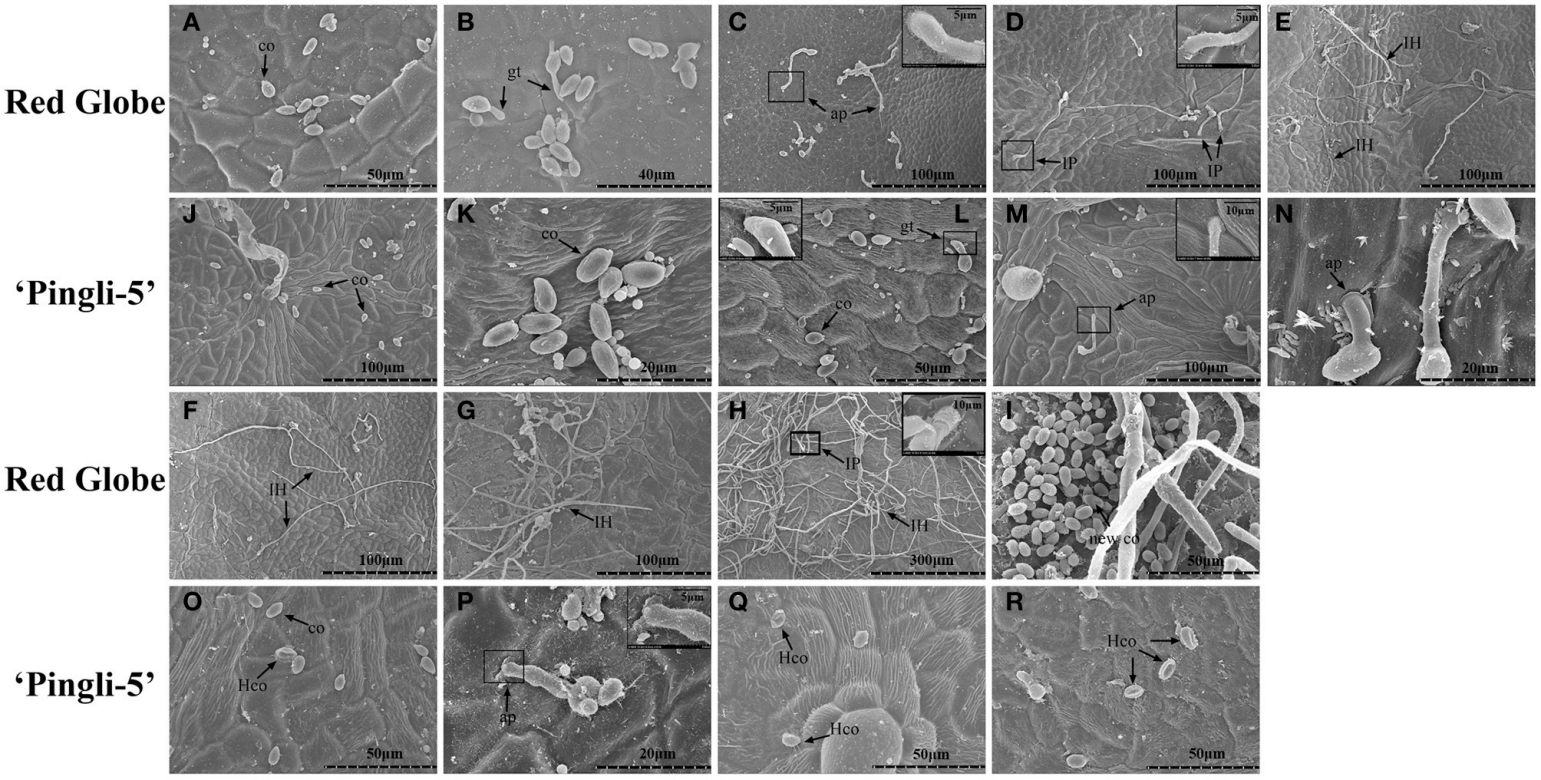
**Comparison of *B. cinerea* conidia development on “Pingli-5” and “Red Globe” leaves using scanning electron microscopy**. Progression of *B. cinerea* colonization on “Red Globe” **(A–I)** and “Pingli-5” **(J–R)**. Leaves were harvested 4, 8, 12, 18, 24, 36, 48, 72, and 96 h post-inoculation (hpi) and the experiments were repeated three times. Arrows indicate a co, conidium; gt, germ tube; ap, appressorium; IP, infection peg; IH, infection hypha; new co, new conidium; and Hco, hollow conidium. Large black blocks indicate magnifications at the sites of small black blocks. Scale bars: **(A,I,L,O,Q,R)**: 50 μm; **(B)**: 40 μm; **(C–G,J,M)**: 100 μm; **(H)**: 300 μm; **(K,N,P)**: 20 μm; Magnification pictures in **(C,D, L,P)**: 5 μm; Magnification pictures in **(H,M)**: 10 μm.

The SEM time series observations indicated that the infection of “Red Globe” was more substantial and aggressive (Figures 4A–I), while the germination was delayed and fungal growth was mostly blocked on “Pingli-5” leaves at the early time points of the initial 24 h infection (Figures 4J–R). No difference was observed 4 hpi (Figures 3B, 4A,J). On “Red Globe,” germination rate increased rapidly to 39% 8 hpi when appressoria were observed (Figure 4B) and to 47% 12 hpi when penetrations were apparent (Figure 4C), after that, infection rate increased to 30% 18 hpi when infection pegs were apparent (Figures 3B, 4D). Then, infection rate increased to 38% 24 hpi, while germination increased slowly (Figure 3B), and this was accompanied by germ tube elongation and the appearance of necrotic spots (Figures 3, 4). During this period, *B. cinerea* failed to capture “Pingli-5” (Figures 4K–N) and germination and infection rates were far lower than “Red Globe” (Figure 3B). The presence of appressoria surrounded by sheaths was first noted 18 hpi (Figure 4M) which seemed to peel away from leaf surfaces (Figures 4N,P), suggesting an even lower rate of infection on “Pingli-5” than that was observed by light microscopy. Infections on “Pingli-5” increased slowly with 6% 18 hpi and 10% 48 hpi (Figure 3B). From 24 hpi, *B. cinerea* germination, and infection on “Red Globe” leaves increased steadily until 96 hpi (Figure 3B). Many hyphae branched (Figure 4G), and a collapse of plant cells around infection sites (Figures 4E,F) and obvious lesion spreading accompanied. From 48 hpi onwards, the fungus grew rudely and sporulated on “Red Globe” (Figures 4G,I). In contrast, *B. cinerea* growth was blocked at an early stage on “Pingli-5” and subsequently the infection was almost completely abolished (Figures 4N–R). The hollow conidia described above were present as early as 36 hpi (Figures 4A–O) and were observed in increasing numbers until 96 hpi (Figures 4A–R).

### H_2_O_2_ accumulation in the interactions of *B. cinerea* with HS “red globe” and HR chinese wild *Vitis* “pingli-5”

**Figure 5:**
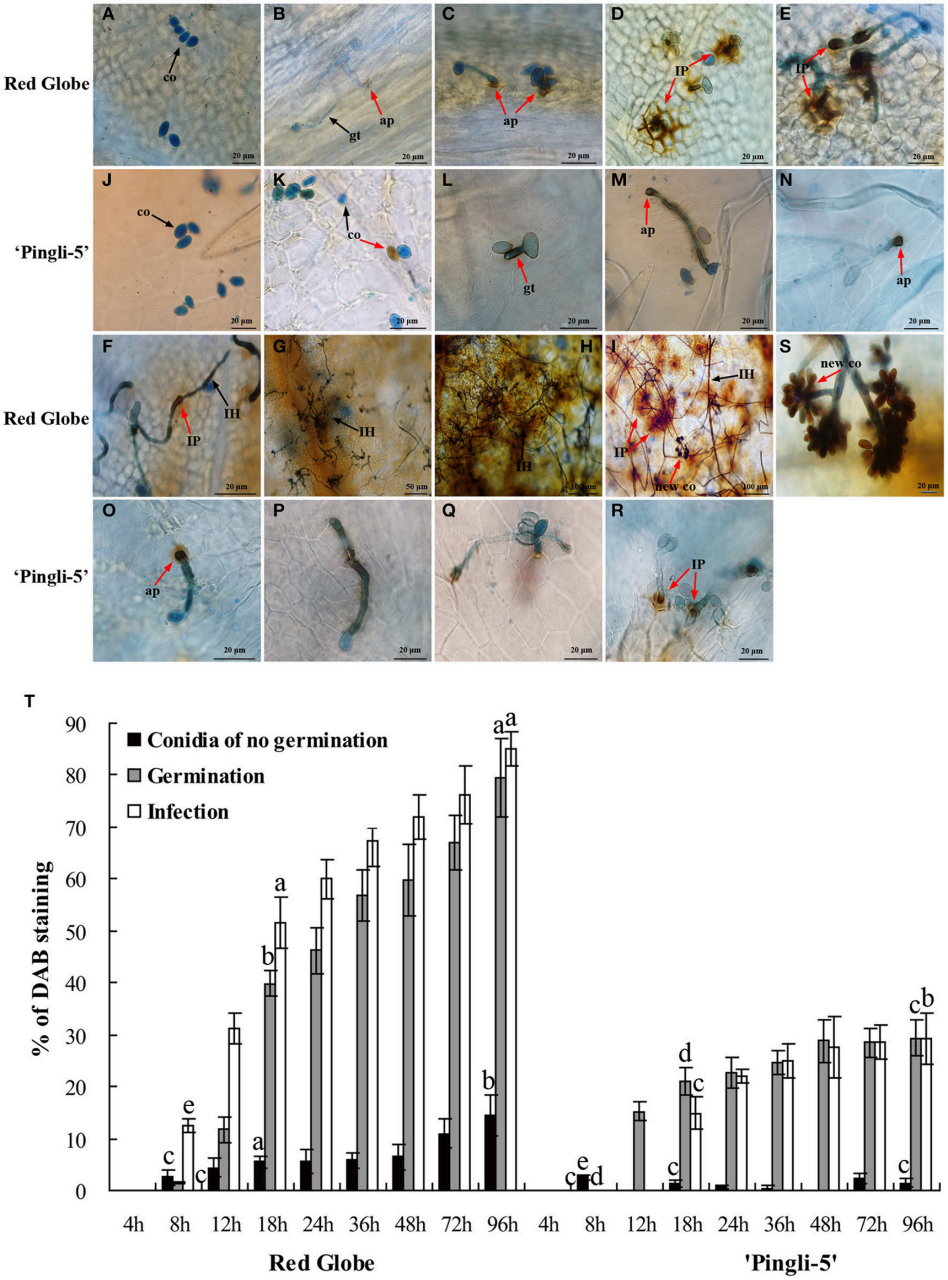
**Temporal evolution of H_2_O_2_ accumulation in the leaves of “Red Globe” and “Pingli-5” as well as in *B. cinerea* following inoculation**. Aniline blue stains the fungus a bluish color while DAB (3-diaminobenzidine) stains H_2_O_2_ purple. H_2_O_2_ accumulation was assessed in the interactions of “Red Globe” **(A–I)** and “Pingli-5” **(J–R)** with *B. cinerea* 4, 8, 12, 18, 24, 36, 48, 72, and 96 h post-inoculation (hpi). Arrows indicate a co, conidium; gt, germ tube; ap, appressorium; IP, infection peg; IH, infection hypha; and new co, new conidium. Black arrows indicate no DAB staining and red arrows indicate DAB staining. **(S)** Higher magnification of the site of the red arrow in **(I)**, showing the DAB stained sporulation. Scale bars: **(A–F)**: 20 μm; **(G)**: 50 μm; **(H,I)**: 100 μm; **(J–R)**: 20 μm; **(S)**: 5 μm. **(T)** Percentages of *B. cinerea* conidia, germ tubes and infection sites exhibiting H_2_O_2_ accumulation at the indicated times. At least 300 conidia were counted at each time point. Experiments were repeated three times with similar results. Bars represent standard deviations. Comparisons of statistical significance were made for the three indices. Different small alphabetical letters indicate statistically significant differences between different interactions of “Red Globe” and “Pingli-5” with *B. cinerea* at the indicated time points (Duncan's multiple range test; *P* < 0.05).

Since one of the earliest defense responses in plant—*B. cinerea* interactions is ROS production (van Kan, 2006; Asselbergh et al., 2007), H_2_O_2_ accumulation was measured during the interactions of *B. cinerea* with HS “Red Globe” and HR Chinese wild *Vitis* “Pingli-5” through DAB staining: brown precipitates at the sites of H_2_O_2_ accumulation due to DAB polymerization (ThordalChristensen et al., 1997). “Red Globe” and “Pingli-5” leaves were sampled 4, 8, 12, 18, 24, 36, 48, 72, and 96 hpi. No staining or germination was observed 4 hpi with either genotype (Figures 5A,J). H_2_O_2_ accumulation was evident 12 hpi in “Red Globe” epidermal cell walls that were in close contact with 31% of the infecting appressoria (Figure 5T), and was also observed in the interspaces between appressoria and epidermal cell walls (Figure 5C). From 12 to 18 hpi, H_2_O_2_ accumulation expanded from the sites of fungal contact, resulting in intense DAB staining in all epidermal cell walls surrounding approximately 55% of the infection sites (Figures 5D,T). Intracellular H_2_O_2_ also accumulated adjacent to “Red Globe” epidermal cell walls (Figure 5D). None of these reactions were visible in “Pingli-5” at these early time points (Figures 5K–M).

H_2_O_2_ generation in *B. cinerea* conidia, germinating spores, and infection structures was also indicated by DAB staining from 8 hpi onwards (Figure 5T), with gradual increases observed over time. However, much lower values were detected for “Pingli-5.” On “Red Globe,” low levels of DAB staining were detected 8 hpi in approximately 13% of the appressoria (Figures 5B,T), and subsequently, H_2_O_2_ accumulation increased at fungal infection sites with an increase of 34% on “Red Globe.” Instead, H_2_O_2_ accumulation was apparent in or around 21% of the germ tubes and 15% of the initial appressoria on “Pingli-5” (Figures 5M,T). On both genotypes, H_2_O_2_ accumulation was observed from 8 hpi onwards, with the largest changes from 8 to 18 hpi: with an increase of 39% on “Red Globe,” and 15% on “Pingli-5” (Figure 5T). DAB staining was especially strong in the top ends of germ tubes and appressoria associated with infection sites, and was much stronger on “Red Globe” Figures 5C,D) than “Pingli-5” (Figures 5K–M).

From 18 to 48 hpi, the extent of H_2_O_2_ distribution in the epidermal cells of “Red Globe” decreased gradually and more intense DAB staining was detected in the infection pegs, the elongating and branching hyphae as necrosis spread (Figures 5E–G). At later time points, during the period of cell death and rapid rot of “Red Globe” leaves, DAB staining of extracellular, and intracellular plant tissue, as well as *B. cinerea* sporulation structures, was very intense (Figures 5H,I). In contrast, appressoria associated with infection sites on “Pingli-5” exhibited increased H_2_O_2_ accumulation of only about 7%. Even though some appressoria on “Pingli-5” were strongly stained, only a few successful infections and limited H_2_O_2_ accumulation at the infection sites were observed (Figures 5N–R).

### O${}_{2}^{-}$ accumulation in the interactions of *B. cinerea* with HS “red globe” and HR chinese wild *Vitis* “pingli-5”

**Figure 6:**
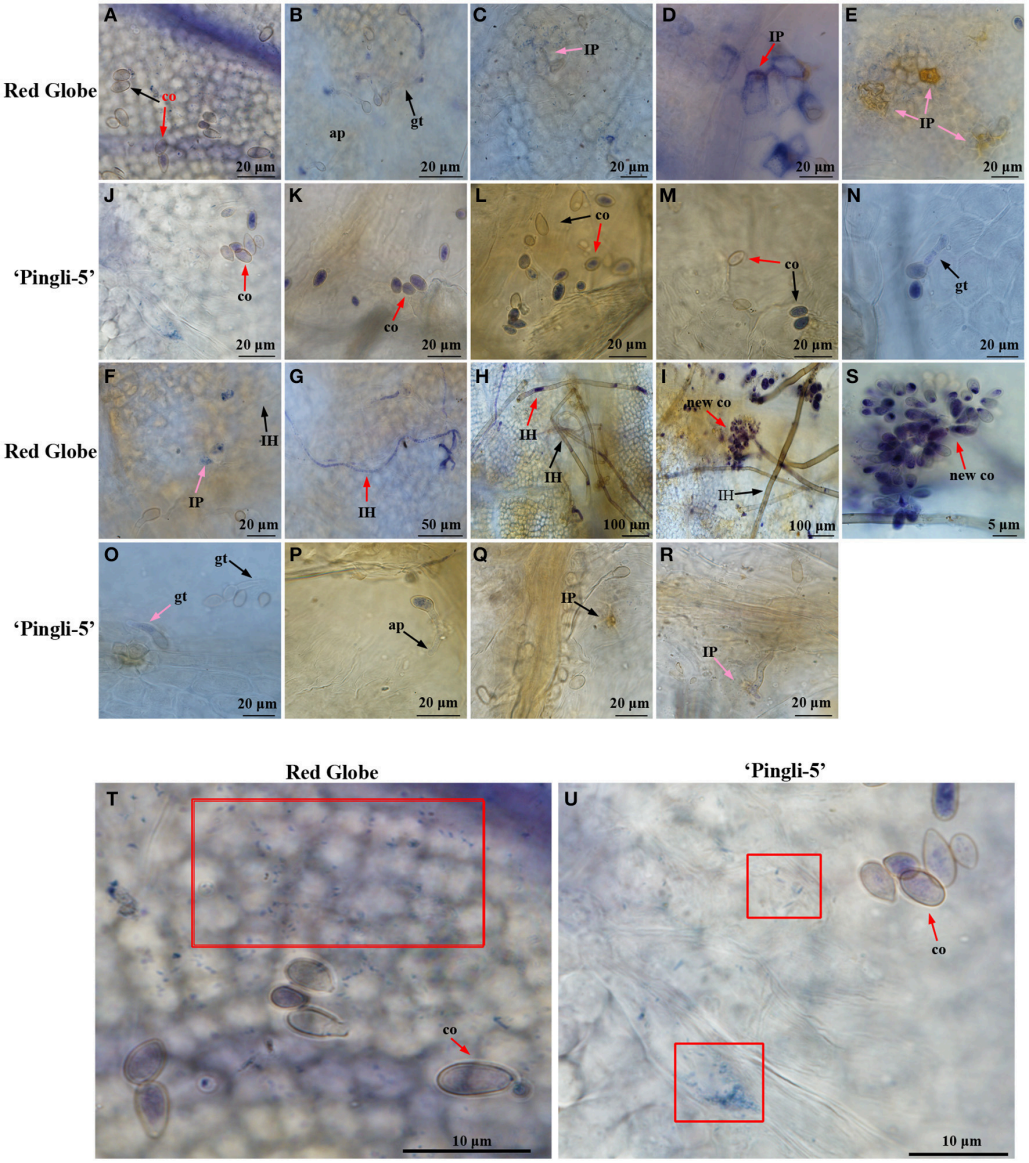
**Temporal evolution of O${}_{2}^{-}$ accumulation in the leaves of “Red Globe” and “Pingli-5” as well as in *B. cinerea* following inoculation**. Nitroblue tetrazolium (NBT) stains O${}_{2}^{-}$ purple and was used to assess O${}_{2}^{-}$ accumulation in the interactions between *B. cinerea* and “Red Globe” **(A–I)** or “Pingli-5” **(J–R)** with *B. cinerea* 4, 8, 12, 18, 24, 36, 48, 72, and 96 h post-inoculation (hpi). Arrows indicate a co, conidia; gt, germ tube; ap, appressorium; IP, infection peg, IH, infection hypha; and new co, new conidium. Black arrows indicate no NBT staining and red arrows indicate means DAB staining. **(S)** Higher magnification of the site of the red arrow in **(I)**, showing the NBT stained sporulation. **(T,U)** O${}_{2}^{-}$ accumulation 4 hpi in the leaves of “Red Globe” **(T)** and “Pingli-5” **(U)** infected with *B. cinerea*. Red blocks indicate wispy and small NBT stained spots in cells of both *Vitis* genotypes. Arrows indicate a NBT stained conidium (co). Scale bar: **(A–F)**: 20 μm; **(G)**: 50 μm; **(H,I)**: 100 μm; **(J–R)**: 20 μm; **(S)**: 5 μm; **(T,U)**: 10 μm.

The accumulation of O${}_{2}^{-}$ was assessed by NBT staining (Wang et al., 2007), which forms a bluish violet precipitate at the sites of O${}_{2}^{-}$ accumulation. Leaf samples of HS “Red Globe” and HR Chinese wild *Vitis* “Pingli-5” were collected at the indicated time points. O${}_{2}^{-}$ generation indicated by small wispy spots of NBT staining occurred over larger areas in “Red Globe” (Figure 6T) than in “Pingli-5” 4 hpi (Figure 6U) whether conidia were present or not. These almost disappeared in “Pingli-5” from 8 hpi onwards (Figures 6K–R). The patterns of O${}_{2}^{-}$ accumulation in “Red Globe” from 8 hpi onwards were very different from those 4 hpi: the *B. cinerea*-”Red Globe” interactions resulted in dark and concentrated NBT staining in the epidermal cell walls in close contact to 47% of infection appressoria, and in the interspaces of epidermal cells and appressoria (Figure 6B). By 12 hpi, O${}_{2}^{-}$ accumulation weakened in the majority of infection appressoria and the epidermal cells around 74% of them when infection sites formed (Figure 6C). However, by 18 hpi, the spreading of O${}_{2}^{-}$ accumulation from the sites of fungal contact resulted in more intense NBT staining of the entire cell walls of many layers of cells around about 73% of the infection sites (Figure 6D and Figure S2); however, O${}_{2}^{-}$ accumulation in “Red Globe” cells declined 24 hpi and was absent 36 hpi (Figures 6E–I), while O${}_{2}^{-}$ accumulated rapidly in infection pegs, hyphae, mycelium, and new sporulation from 36 to 96 hpi (Figures 5E–G). None of these reactions in “Red Globe” from 8 hpi was observed in “Pingli-5” (Figures 6J–R). Contrastingly, the proportion of conidia that did not germinate but showed NBT staining increased 8 hpi following a gruadually decline until 96 hpi. At last, 18% infection structures showed NBT staining (Figure S2) and “Pingli-5” cells beneath these infection sites showed only limited and indistinct staining (Figures 6Q,R).

The percentages of *B. cinerea* conidia, germinating spores, and infection sites generating O${}_{2}^{-}$ were analyzed in “Pingli-5” and “Red Globe” (Figure S2). These three indices from “Pingli-5” decreased, except for that NBT staining conidia increased marginally from 90% 4 hpi to 96% 8 hpi and that germinating spores with NBT staining increased from 26% 8 hpi to 35% 12 hpi. In the case of “Red Globe,” conidia showing NBT staining decreased from 92% 4 hpi to the lowest percent of 29% 72 hpi, and then increased to 39% 96 hpi as a consequence of new sporulation. Germination conidia showing NBT staining first occurred with the percent of 73% 8 hpi and declined to the lowest percent of 51% 48 hpi, before increasing to 74% 96 hpi. A total of 67% infection sites with NBT staining first appeared 8 hpi and 74% showed staining 24 hpi, before the number decreased to 55% 36 hpi and increased again to 72% 96 hpi.

### Activities of peroxidase, catalase, and superoxide dismutase in HS “red globe” and HR chinese wild *Vitis* “pingli-5” infected by *B. cinerea*

**Figure 7:**
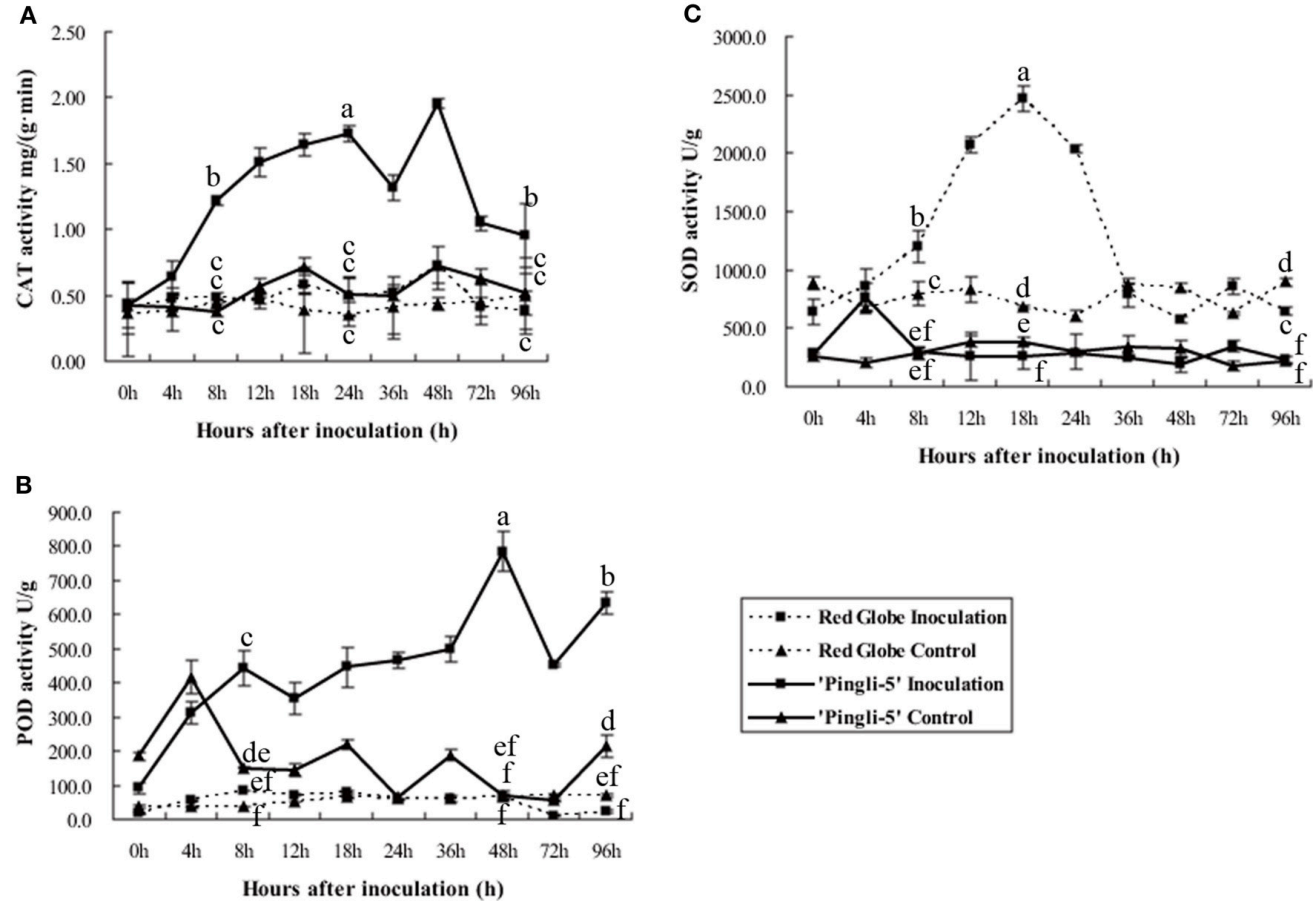
**Activities of catalase (CAT; A), superoxide dismutase (SOD; B) and peroxidases (POD; C) in proteins extracts from “Red Globe” and “Pingli-5” leaves 4, 8, 12, 18, 24, 36, 48, 72, and 96 h post-inoculation (hpi) with *B. cinerea* and sterile water as the control**. The means and standard deviations of three independent experiments are shown. Comparisons of statistical significance were made for the three indices. Different small alphabetical letters indicate statistically significant differences between different interactions of “Red Globe” and “Pingli-5” with *B. cinerea* at the indicated time points (Duncan's multiple range test; *P* < 0.05).

Antioxidant enzymes protect plants from oxidative stress and maintain redox equilibria through scavenging of ROS produced during pathogen attack (Pallavi Sharma et al., 2012). Peroxidase (POD), catalase (CAT) and superoxide dismutase (SOD) activity levels in the leaves of HR “Pingli-5” and HS “Red Globe” were tested to assess the dynamics of the antioxidant system following challenge with *B. cinerea*. Protein extracts from leaves of “Pingli-5” control as well as “Red Globe” inoculation and control all exhibited similar CAT or POD background activities with basical invariant (Figures 7A,B). However, in inoculated “Pingli-5” leaves, CAT activity gradually increased to approximately three-fold the background value 24 hpi, followed by a small drop 36 hpi with another four-fold increase 48 hpi compared to the background value and by 96 hpi, the activity decreased to a value two-fold higher than that of the background (Figure 7A); POD activity increased to a peak of eight-fold higher activity than the background value 48 hpi, followed by a decrease 72 hpi and a final increase of about six times higher than the background 96 hpi (Figure 7B).

Interestingly, background SOD activity in “Red Globe” was approximately twice that of “Pingli-5” in the control assays (Figure 7C). Moreover, SOD activity in inoculated “Pingli-5” leaves was similar to that of the control, except for an almost three-fold increase 4 hpi to nearly the same value with the background activity in “Red Globe” leaves (Figure 7C). In contrast, SOD activity in “Red Globe” leaves increased following *B. cinerea* infection to a maximum of 3.6-fold that of the background activity 18 hpi, but then decreased rapidly to the background levels by 36 hpi with no further increases detected (Figure 7C).

## Discussion

### Chinese wild *Vitis* species represent valuable *B. cinerea* resistant germplasm

It has been reported that most popular *V. vinifera* berries are susceptible to *B. cinerea*, while *V. rotundifolia, V. labrusca*, or other complex hybrids are highly resistant (Gabler et al., 2003). Although *B. cinerea* predominantly infects grape flowers, leaves are the second most infected organs (Holz et al., 2003; van Kan, 2006). To our knowledge, the current study is the first to document the resistance of Chinese wild *Vitis* to *B. cinerea* where leaf resistance levels of 41 genotypes, including 30 Chinese wild *Vitis* species, were described (Tables 1, 2). Intraspecific variation was found since the resistance levels did not correlate perfectly with individual species (Figure 1). Eighteen of the 30 Chinese wild *Vitis* were resistant to the fungus, while little or no resistance was seen in most *V. vinifera* and its hybrids such as “Red Globe,” “Muscat Hamburg,” “No. 8 Hutai” and “Kyoho” (Table 1). Indeed, the six most highly resistant genotypes with extremely less lesion events and relatively low infection rates were all Chinese wild *Vitis* species: three *V. amurensis* Rupr; one ‘*V. yenshanensis*; one *V.* sp. (Qinling grape), and one *V. adstricta* (Table 1 and Figure 2).

Wang et al. (1995, 1998) described Chinese wild *Vitis* as a valuable resource for future disease resistance breeding programs. Many Chinese wild *Vitis* species exhibit synchronous multi-fungal disease defense: *V. amurensis* is known for its tolerance of cold and anthracnose, as a multi-resistant rootstock (Liu and Li, 2013); “Pingli-5” of *V.* sp. (Qinling grape) is resistant to anthracnose, powdery and downy mildew (Wang et al., 1995, 1998). Consequently, Chinese wild *Vitis* of high resistance to *B. cinerea* like “Shuangyou,” “Tonghua-3,” and “Pingli-5” may have the potential to decrease the gray mold in vineyards and protected grapevine cultivation systems, and may therefore represent valuable germplasm for breeding new varieties with resistance to multiple fungal diseases.

### *B. cinerea* growth is blocked in the early infection stages on the highly resistant chinese wild *Vitis* “pingli-5”

In the present study, the distinct colonization of *B. cinerea* on grapevine leaves was first revealed by SEM over a time series. On “Red Globe,” penetration of *B. cinerea* was direct and the pathogen established a primary restricted infection as necrosis occurred before 24 hpi (Figures 3, 4). Subsequently, *B. cinerea* initiated a massive outgrowth and sporulation (Figures 3, 4). Conversely, during the early infection steps before 24 hpi, penetration on “Pingli-5” showed a substantial delay resulting in markedly lower germination and infection rates (Figures 3B, 4). Most appressorium on “Pingli-5” leaves had a sheath (Figure 4) possibly composed of disassembled polysaccharides and secondary metabolites (Viret et al., 2004; van Kan, 2006; Choquer et al., 2007), but they rarely developed into infection pegs like those present on “Red Globe” leaves (Figure 4). Therefore, it seems that the colonization of *B. cinerea* was blocked on Chinese wild *Vitis* “Pingli-5” during these early infection stages, possibly due to its physical and chemical barriers such as cell wall reinforcement and phytoalexin synthesis (Elad, 1997; Adrian and Jeandet, 2012; Cheng et al., 2012) or defense responses such as the timely deployment of ROS (Foyer and Noctor, 2013).

### Reactive oxygen species and antioxidative activities were differentially induced depending on the susceptibility of the *Vitis* genotype to *B. cinerea* infection

After establishing that HR Chinese wild *Vitis* “Pingli-5” could effectively block *B. cinerea* and that HS “Red Globe” was a favorable host, the underlying possible mechanisms of resistance in “Pingli-5” and susceptibility in “Red Globe” were investigated. Since ROS are implicated in plant responses to pathogen attacks (Torres et al., 2006; Foyer and Noctor, 2013) and a detailed time point series evaluation of ROS accumulation during the interactions with *B. cinerea* were conducted, and the potential participation of antioxidant enzymes were assessed.

It has been previously shown that H_2_O_2_ induced in plant cells, accompanied by O${}_{2}^{-}$ generation, can promote programmed cell death in the host and expansion of disease lesions to facilitate *B. cinerea* infection (Govrin and Levine, 2000; Patykowski, 2006; Asai and Yoshioka, 2009; Simon et al., 2013; Zhang et al., 2014). Other studies with *A. thaliana*, tomato and other plants species (Asselbergh et al., 2007; L'Haridon et al., 2011; Windram et al., 2012; Serrano et al., 2014) have also suggested the importance of increased ROS levels in defense against *B. cinerea*. Elicitors and bacteria have been shown to contribute to the ROS based defense mechanism in grapevines (Aziz et al., 2004; Varnier et al., 2009; Verhagen et al., 2010, 2011; Benikhlef et al., 2013). Here, ROS accumulation was not observed in control leaves (data not shown). Overall, high levels of ROS accumulated in the host-fungal interfaces, infection structures, and many layers of epidermal cells surrounding the infection sites between 8 and 18 hpi when infection initiated on HS “Red Globe” (Figures 4–6). Then ROS accumulated continuously in “Red Globe” and *B. cinerea* concurrent with the infection progression and lesion spreading. Conversely, only consistently low levels of ROS accumulation were observed following inoculation of resistant “Pingli-5” with *B. cinerea* (Figures 5, 6). Therefore, it seems that the reliably high level of ROS production seen in “Red Globe” could, at least in part, promote its susceptibility to *B. cinerea* infection and colonization, while the weak ROS induction seen following B. cinerea inoculation of “Pingli-5” may contribute to its resistance.

With regards to antioxidant activity, we found that “Red Globe” leaves inoculated with *B. cinerea* exhibited little change in CAT and POD activities as lesions spread. However, they did display increased SOD activity between 8 and 18 hpi (Figure 7), which correlates well with the increase in H_2_O_2_ levels and diminishment of O${}_{2}^{-}$ from 24 hpi onwards (Figures 5, 6). However, CAT and POD activities in resistant “Pingli-5” increased throughout the experiment, but virtually no change in SOD activity was observed with the exception of an increase 4 hpi (Figure 7), which was consistent with its minimal induction of ROS (Figures 5, 6). Antioxidative systems are critical for controlling timing and strength of ROS production to maintain redox homeostasis (Torres et al., 2006; Mittler et al., 2011) and for protecting cells from ROS damage (Pallavi Sharma et al., 2012). It has been reported that after *B. cinerea* infection, *A. thaliana* (Govrin and Levine, 2000; Simon et al., 2013) and tomato (Asselbergh et al., 2007; Zhang et al., 2014) and *Phaseolus vulgaris* (Muckenschnabel et al., 1954) continuously accumulate ROS and lesions develop for their insufficient antioxidative systems, and it is nessecery that plants timely modulated its own ROS accumulation to low levels through antioxidative system to maintain redox equilibrium (Mittler et al., 2011; Foyer and Noctor, 2013). In line with this, we found that when challenged by *B. cinerea*, susceptible “Red Globe” indeed experienced the effects of an insufficient antioxidative system, resulting in consistently high ROS levels, while “Pingli-5” rapidly upregulated its antioxidative capacity following inoculation (particularly CAT and POD activities) and thus experienced less ROS-induced stress. Since substantial ROS was induced in “Red Globe” but not in “Pingli-5,” the precise coordination of ROS production and associated scavenging mechanisms by antioxidative system during combined biotic and abiotic stress (Atkinson and Urwin, 2012) is likely to be important for Chinese wild *Vitis* “Pingli-5” to defense itself against *B. cinerea*.

It has been proved *B. cinerea* itself also generates ROS (Rolke et al., 2004) and adapts this high oxidative stress (Choquer et al., 2007; Temme and Tudzynski, 2009) but perturbs the redox status in and around the infected tissue, thereby promoting infection, which is important for pathogenicity (van Kan, 2005, 2006). We observed ROS accumulation within the pathogen *B. cinerea* on both grapevine leaves, higher in fungi present on “Red Globe” than “Pingli-5.” In any case, regardless of whether the low antioxidative capacity in “Red Globe” was inherent or caused by the infecting *B. cinerea*, it is clear that “Red Globe” suffered seriously from its sustained ROS accumulation. Instead, “Pingli-5” did not have to contend with huge oxidative stress for its highly and timely elevated antioxidative capacity.

Much attention has been paid to H_2_O_2_ induction in plants, which has been conflictingly found to contribute to either increased resistance or susceptibility toward *B. cinerea*, and on the other hand, O${}_{2}^{-}$ has generally been suggested to act as a primary substrate to form H_2_O_2_ (Govrin and Levine, 2000; Torres et al., 2006; van Kan, 2006; Asselbergh et al., 2007; Serrano et al., 2014). Some reports have suggested that O${}_{2}^{-}$ plays a role in promoting *B. cinerea* invasion (Urbanek et al., 1996; Patykowski, 2006; Zhang et al., 2014); however in studies of infected and mock infected tomato leaves, no O${}_{2}^{-}$ accumulation was observed (Asselbergh et al., 2007). In bean, the induction of O${}_{2}^{-}$ production in leaves is thought to be one of the key factors that differentiate the interactions with the compatible and incompatible pathogens: *B. fabae* and *B. cinerea*, respectively (Urbanek et al., 1996). Furthermore, it has been proposed that if strong oxidative damage at an early stage is insufficient to arrest the pathogen, its subsequent development will be less sensitive to oxidizing agents and so a relatively weak oxidative burst may serve to promote antioxidant systems, ultimately increasing its tolerance to subsequent oxidative stress (Gessler et al., 2007). Here, O${}_{2}^{-}$ accumulating was detected ealier than H_2_O_2_ in inoculated leaves of both hosts (with or without conidia on). This accumulation began 4 hpi, at the earliest stage of the infection (Figures 6T,U), but was present to a lesser extent in the highly resistant “Pingli-5” and all disappeared from 8 hpi onwards in “Pingli-5” when more O${}_{2}^{-}$ begun to accumulate around the infection sites in “Red Globe” (Figure 6). At this same time point, O${}_{2}^{-}$ also accumulated in more than 85% of conidia themselves on both hosts (Figure 6), which was also earlier than H_2_O_2_ production began within the fungus (Figure 5).

Taken together, we assume that at the earliest stages of the different interaction systems, similar O${}_{2}^{-}$ levels generated by *B. cinerea* may provide the same attack signal both to “Red Globe” and “Pingli-5,” but could induce distinct O${}_{2}^{-}$ responses in hosts. This might in turn effect subsequent ROS accumulation, antioxidative system levels and infection progression. An induction of O${}_{2}^{-}$ generation, earlier than H_2_O_2_ production, may be among the first consequences of an interaction between *B. cinerea* with grapevines. The higher levels of O${}_{2}^{-}$ induced in HS “Red Globe” compared to “Pingli-5” at the earliest infection stages (before 8 hpi) could potentially result in much higher and sustained ROS levels with its insufficient antioxidative protection during subsequent infection periods and could ultimately lead to oxidative damage and cell death. In comparison, the lower levels of O${}_{2}^{-}$ in “Pingli-5” at the earliest infection stages (before 8 hpi) may represent a low/moderate concentration for a recognition process for timely elevating antioxidative capacity to prevent the subsequent sustained ROS production and arrest the attachment and development of *B. cinerea*. However, at present, this is a simply conjecture and would require further research to provide definitive answers with regards to the importance of the timing of O${}_{2}^{-}$ and H_2_O_2_ accumulation. Thus, the spatiotemporal relationship between ROS and antioxidative systems and other signaling molecules remains an interesting area to better understand the resistance of Chinese wild *Vitis* against *B. cinerea* and allow the development of *B. cinerea* resistant grapes.

In conclusion, we explored germplasm resources from Chinese wild *Vitis* species for resistance to *B. cinerea* that causes the gray mold disease. A lack of resistance in most cultivated genotypes was confirmed and a substantial amount of resistance in Chinese wild *Vitis* species was identified using detached leaf assays. The events leading to *B. cinerea* resistance in Chinese wild *Vitis* species were further investigated by contrasting fungal growth, reactive oxygen species (ROS) responses and antioxidative system changes between the highly susceptible *Vitis vinifera* “Red Globe” and the highly resistant Chinese wild *Vitis* “Pingli-5” [*V.* sp. (Qinling grape)] after the infection with this pathogen. Our results demonstrated that minimal fungal development as well as minimal production of ROS and a timely elevation in antioxidative capacity were correlated with a high level of resistance in “Pingli-5,” while highly suscepitble “Red Globe” suffered massive infection and sustained ROS production due to relatively unchanged antioxidative activities. Moreover, we speculated O${}_{2}^{-}$ induction, which occurred earlier than H_2_O_2_ production, may be among the first consequences of an interaction between *B. cinerea* with grapevines, suggesting a potential ROS response responsible for the timely recognition and defense of Chinese wild *Vitis* “Pingli-5” to *B. cinerea*. However, this remains to be resolved through futher experiments on spatiotemporal relationship of ROS and molecular mechanism.

## Author contributions

XPW and RW designed the study. RW, XH, and XHW contributed to the experiments. RW, XH, and JQ performed data analysis. RW, XH, and SS assisted with the interpretation of the results. XPW and YW provided guidance throughout the study. RW, XH, SS, and XPW wrote and revised the manuscript. All authors approved the final manuscript.

### Conflict of interest statement

The authors declare that the research was conducted in the absence of any commercial or financial relationships that could be construed as a potential conflict of interest.

## References

[B1] Adrian M. Jeandet P. (2012). Effects of resveratrol on the ultrastructure of *Botrytis cinerea* conidia and biological significance in plant/pathogen interactions. Fitoterapia 83, 1345–1350. 10.1016/j.fitote.2012.04.004 22516542

[B2] Afzal F. Khurshid R. Ashraf M. Kazi A. G. (2014). Reactive Oxygen Species and Antioxidants in Response to Pathogens and Wounding. Oxidative Damage to Plants: Antioxidant Networks and Signaling. London: Elsevier Science press, 397–424.

[B3] Angelini R. M. D. M. Rotolo C. Masiello M. Gerin D. Pollastro S. Faretra F. (2014). Occurrence of fungicide resistance in populations of *Botryotinia fuckeliana* (*Botrytis cinerea*) on table grape and strawberry in southern Italy. Pest Manag Sci. 70, 1785–1796. 10.1002/ps.3711 24338954

[B4] Asai S. Yoshioka H. (2009). Nitric oxide as a partner of reactive oxygen species participates in disease resistance to necrotrophic pathogen *Botrytis cinerea* in *Nicotiana benthamiana*. Mol. Plant Microbe Interact. 22, 619–629. 10.1094/MPMI-22-6-0619 19445587

[B5] Asselbergh B. Curvers K. Franca S. C. Audenaert K. Vuylsteke M. van Breusegem F.. (2007). Resistance to *Botrytis cinerea* in sitiens, an abscisic acid-deficient tomato mutant, involves timely production of hydrogen peroxide and cell wall modifications in the epidermis. Plant Physiol. 144, 1863–1877. 10.1104/pp.107.099226 17573540 PMC1949893

[B6] Atkinson N. J. Urwin P. E. (2012). The interaction of plant biotic and abiotic stresses: from genes to the field. J. Exp. Bot. 63, 3523–3543. 10.1093/jxb/ers100 22467407

[B7] Audenaert K. De Meyer G. B. Höfte M. M. (2002). Abscisic acid determines basal susceptibility of tomato to *Botrytis cinerea* and suppresses salicylic acid-dependent signaling mechanisms. Plant Physiol. 128, 491–501. 10.1104/pp.010605 11842153 PMC148912

[B8] Aziz A. Heyraud A. Lambert B. (2004). Oligogalacturonide signal transduction, induction of defense-related responses and protection of grapevine against B. cinerea. Planta 218, 767–774. 10.1007/s00425-003-1153-x 14618326

[B9] Benikhlef L. L'Haridon F. Abou-Mansour E. Serrano M. Binda M. Costa A.. (2013). Perception of soft mechanical stress in *Arabidopsis* leaves activates disease resistance. BMC Plant Biol. 13:133. 10.1186/1471-2229-13-133 24033927 PMC3848705

[B10] Buxdorf K. Rubinsky G. Barda O. Burdman S. Aharoni A. Levy M. (2014). The transcription factor *SlSHINE3* modulates defense responses in tomato plants. Plant Mol Biol. 84, 37–47. 10.1007/s11103-013-0117-1 23943056

[B11] Cheng Y. Zhang H. Yao J. Wang X. Xu J. Han Q.. (2012). Characterization of non-host resistance in broad bean to the wheat stripe rust pathogen. BMC Plant Biol. 12:96. 10.1186/1471-2229-12-96 22716957 PMC3487988

[B12] Choquer M. Fournier E. Kunz C. Levis C. Pradier J.-M. Simon A.. (2007). *Botrytis cinerea* virulence factors: new insights into a necrotrophic and polyphageous pathogen. Fems Microbiol Lett. 277, 1–10. 10.1111/j.1574-6968.2007.00930.x 17986079

[B13] De Tullio M. C. (2010). Antioxidants and redox regulation: changing notions in a changing world. Plant Physiol Biochem. 48, 289–291. 10.1016/j.plaphy.2010.02.011 20299232

[B14] Elad Y. (1997). Responses of plants to infection by *Botrytis cinerea* and novel means involved in reducing their susceptibility to infection. Biol. Rev. 72, 381–422.

[B15] Foyer C. H. Noctor G. (2013). Redox signaling in plants. Antioxid. Redox Signal. 18, 2087–2090. 10.1089/ars.2013.5278 23442120

[B16] Gabler F. M. Smilanick J. L. Mansour M. Ramming D. W. Mackey B. E. (2003). Correlations of morphological, anatomical, and chemical features of grape berries with resistance to *Botrytis cinerea*. Phytopathology 93, 1263–1273. 10.1094/PHYTO.2003.93.10.1263 18944326

[B17] Gessler N. N. Aver'Yanov A. A. Belozerskaya T. A. (2007). Reactive oxygen species in regulation of fungal development. Biochem. Moscow 72, 1091–1109. 10.1134/S0006297907100070 18021067

[B18] Giannopolitis C. N. Ries S. K. (1977). Superoxide dismutases: I. Occurrence in higher plants. Plant Physiol. 59, 309–314. 10.1104/pp.59.2.309 16659839 PMC542387

[B19] Govrin E. M. Levine A. (2000). The hypersensitive response facilitates plant infection by the necrotrophic pathogen *Botrytis cinerea*. Curr Biol. 10, 751–757. 10.1016/S0960-9822(00)00560-1 10898976

[B20] Holz G. Gutschow M. Coertze S. Calitz F. J. (2003). Occurrence of *Botrytis cinerea* and subsequent disease expression at different positions on leaves and bunches of grape. Plant Dis. 87, 351–358. 10.1094/PDIS.2003.87.4.351 30831828

[B21] Lamb C. Dixon R. A. (1997). The oxidative burst in plant disease resistance. Ann. Rev. Plant Physiol. Plant Mol. Biol. 48, 251–275. 10.1146/annurev.arplant.48.1.251 15012264

[B22] L'Haridon F. Besson-Bard A. Binda M. Serrano M. Abou-Mansour E. Balet F.. (2011). A permeable cuticle is associated with the release of reactive oxygen species and induction of innate immunity. PLoS Pathog. 7:e1002148. 10.1371/journal.ppat.1002148 21829351 PMC3145797

[B23] Liu L. Li H. (2013). Review: research progress in amur grape, *Vitis amurensis* Rupr. Can. J. Plant Sci. 93, 565–575. 10.4141/cjps2012-202

[B24] Liu S. M. Sykes S. R. Clingeleffer P. R. (2003). A method using leafed single-node cuttings to evaluate downy mildew resistance in grapevine. Vitis 42, 173–180. Available online at: http://www.vitis-vea.de/admin/volltext/e049263.pdf

[B25] Luo S. L. He P. H. (2004). The inheritances of fruit skin and must colors, in a series of interspecific and intraspecific crosses between *V vinifera* and the wild grape species native to China. Sci. Horticul. 99, 29–40. 10.1016/S0304-4238(03)00085-2

[B26] lv Q. (2013). Researh on Modern China's Wine Industry Development. Dissertation, Northwest A & F University.

[B27] Maehly A. C. Chance B. (1954). The assay of catalases and peroxidases. Meth. Biochem. Anal. 1, 357–424. 13193536 10.1002/9780470110171.ch14

[B28] Mittler R. Vanderauwera S. Suzuki N. Miller G. Tognetti V. B. Vandepoele K.. (2011). ROS signaling: the new wave? Trends Plant Sci. 16, 300–309. 10.1016/j.tplants.2011.03.007 21482172

[B29] Muckenschnabel I. Williamson B. Goodman B. A. Lydon G. D. Stewart D. Deighton N. (1954). Markers for oxidative stress associated with soft rots in French beans (*Phaseolus vulgaris*) infected by *Botrytis cinerea*. Planta 212, 376–381. 10.1007/s004250000401 11289602

[B30] Pallavi Sharma Jha, A. B. Dubey R. S. Pessarakli M. (2012). Reactive oxygen species, oxidative damage, and antioxidative defense mechanism in plants under stressful conditions. J. Bot. 2012:217037 10.1155/2012/217037

[B31] Patykowski J. (2006). Role of hydrogen peroxide and apoplastic peroxidase in tomato—*Botrytis cinerea* interaction. Acta Physiol. Plant 28, 589–598. 10.1007/s11738-006-0054-6

[B32] Poolsawat O. Tharapreuksapong A. Wongkaew S. Chaowiset W. Tantasawat P. (2012). Laboratory and field evaluations of resistance to *Sphaceloma ampelinum* causing anthracnose in grapevine. Aust. Plant Pathol. 41, 263–269. 10.1007/s13313-012-0127-5

[B33] Rolke Y. Liu S. J. Quidde T. Williamson B. Schouten A. Weltring K. M. (2004). Functional analysis of H_2_O_2_-generating systems in *Botrytis cinerea*: the major Cu-Zn-superoxide dismutase (BCSOD1) contributes to virulence on French bean, whereas a glucose oxidase (BCGOD1) is dispensable. Mol. Plant Path. 5, 17–27. 10.1111/j.1364-3703.2004.00201.x 20565578

[B34] Schumacher J. Tudzynski P. (2012). Morphogenesis and pathogenicity in fungi, in Topics in Current Genetics, eds Peérez-Martín J. Di Pietro A. (Heidelberg: Springer), 225–241.

[B35] Serrano M. Coluccia F. Torres M. L'Haridon F. Metraux J.-P. (2014). The cuticle and plant defense to pathogens. Front. Plant Sci. 5:274. 10.3389/fpls.2014.00274 24982666 PMC4056637

[B36] Simon U. K. Polanschütz L. M. Koffler B. E. Zechmann B. (2013). High resolution imaging of temporal and spatial changes of subcellular ascorbate, glutathione and H_2_O_2_ distribution during *Botrytis cinerea* infection in *Arabidopsis*. PLoS ONE 8:e65811 10.1371/journal.pone.0065811 23755284 PMC3673919

[B37] Temme N. Tudzynski P. (2009). Does *Botrytis cinerea* ignore H_2_O_2_-induced oxidative stress during infection? characterization of *Botrytis* activator protein 1. Mol. Plant Microbe Interact. 22, 987–998. 10.1094/MPMI-22-8-0987 19589074

[B38] ThordalChristensen H. Zhang Z. G. Wei Y. D. Collinge D. B. (1997). subcellular localization of H_2_O_2_ in plants. H_2_O_2_ accumulation in papillae and hypersensitive response during the barley-powdery mildew interaction. Plant J. 11, 1187–1194. 10.1046/j.1365-313X.1997.11061187.x

[B39] Tierens K. Thomma B. Bari R. P. Garmier M. Eggermont K. Brouwer M.. (2002). Esa1, an *Arabidopsis* mutant with enhanced susceptibility to a range of necrotrophic fungal pathogens, shows a distorted induction of defense responses by reactive oxygen generating compounds. Plant J. 29, 131–140. 10.1046/j.1365-313x.2002.01199.x 11862946

[B40] Torres M. A. Jones J. D. G. Dangl J. L. (2006). Reactive oxygen species signaling in response to pathogens. Plant Physiol. 141, 373–378. 10.1104/pp.106.079467 16760490 PMC1475467

[B41] Urbanek H. Gajewska E. Karwowska R. Wielanek M. (1996). Generation of superoxide anion and induction of superoxide dismutase and peroxidase in bean leaves infected with pathogenic fungi. Acta Biochim. Pol. 43, 679–685. 9104504

[B42] Vandelle E. Poinssot B. Wendehenne D. Bentéjac M. Alain P. (2006). Integrated signaling network involving calcium, nitric oxide, and active oxygen species but not mitogen-activated protein kin in BcPG1-elicited grapevine defenses. Mol. Plant Microbe Interact. 19, 429–440. 10.1094/MPMI-19-0429 16610746

[B43] van Kan J. A. L. (2005). Infection strategies of *Botrytis cinerea*, in Proceedings of the Viiith International Symposium on Postharvest Physiology of Ornamental Plants, Vol. 669, eds Marissen N. VanDoorn W. G. VanMeeteren U. (Wageningen: Acta Hortic, Pressed in Doorwerth), 77–89. 10.17660/ActaHortic.2005.669.9

[B44] van Kan J. A. L. (2006). Licensed to kill: the lifestyle of a necrotrophic plant pathogen. Trends Plant Sci. 11, 247–253. 10.1016/j.tplants.2006.03.005 16616579

[B45] Varnier A.-L. Sanchez L. Vatsa P. Boudesocque L. Garcia-Brugger A. Rabenoelina F.. (2009). Bacterial rhamnolipids are novel MAMPs conferring resistance to *Botrytis cinerea* in grapevine. Plant Cell Environ. 32, 178–193. 10.1111/j.1365-3040.2008.01911.x 19021887

[B46] Verhagen B. Trotel-Aziz P. Jeandet P. Baillieul F. Aziz A. (2011). Improved Resistance Against *Botrytis cinerea* by Grapevine-Associated Bacteria that induce a prime oxidative burst and phytoalexin production. Phytopathology 101, 768–777. 10.1094/PHYTO-09-10-0242 21425931

[B47] Verhagen B. W. M. Trotel-Aziz P. Couderchet M. Höefte M. Aziz A. (2010). *Pseudomonas* spp.-induced systemic resistance to *Botrytis cinerea* is associated with induction and priming of defence responses in grapevine. J. Exp. Bot. 61, 249–260. 10.1093/jxb/erp295 19812243

[B48] Viret O. Keller M. Jaudzems V. G. Cole F. M. (2004). *Botrytis cinerea* infection of grape flowers: light and electron microscopical studies of infection sites. Phytopathology 94, 850–857. 10.1094/PHYTO.2004.94.8.850 18943105

[B49] Wang C.-F. Huang L.-L. Buchenauer H. Han Q.-M. Zhang H.-C. Kang Z.-S. (2007). Histochemical studies on the accumulation of reactive oxygen species (O^−^_2_ and H_2_O_2_) in the incompatible and compatible interaction of wheat—*Puccinia striiformis* f. sp. *tritici*. Physiol. Mol. Plant P. 71, 230–239. 10.1016/j.pmpp.2008.02.006

[B50] Wang Y. Liu Y. He P. Chen J. Lamikanra O. Lu J. (1995). Evaluation of foliar resistance to *Uncinula Necator* in Chinese wild *Vitis* species. Vitis 34, 159–164.

[B51] Wang Y. Liu Y. He P. Lamikanra O. Lu J. (1998). Resistance of Chinese *Vitis* species to *Elsinoe ampelina* (de Bary) Shear. Hortscience 33, 123–126.

[B52] Windram O. Madhou P. McHattie S. Hill C. Hickman R.. (2012). *Arabidopsis* Defense against *Botrytis cinerea*: chronology and regulation deciphered by high-resolution temporal transcriptomic analysis. Plant Cell 24, 3530–3557. 10.1105/tpc.112.102046 23023172 PMC3480286

[B53] Zhang P. (2011). Study on Occuring Rugularity and Control Techniques of Grape Gray Mould. Dissertation, Chinese Academy of Agricultural Sciences.

[B54] Zhang Y. Liu B. Li X. Ouyang Z. Huang L. Hong Y.. (2014). The de novo biosynthesis of vitamin B6 Is required for disease resistance against *Botrytis cinerea* in tomato. Mol. Plant Microbe Interact. 27, 688–699. 10.1094/MPMI-01-14-0020-R 24678833

