# Supplementary material for: Resistance evaluation of Chinese wild Vitis genotypes against Botrytis cinerea and different responses of resistant and susceptible hosts to the infection
Source: Front Plant Sci. 2015 Oct 26;6:854. doi: 10.3389/fpls.2015.00854 (PMC4620147; doi:10.3389/fpls.2015.00854)
Supplement: Supplementary file 2 [file Image1.PDF]

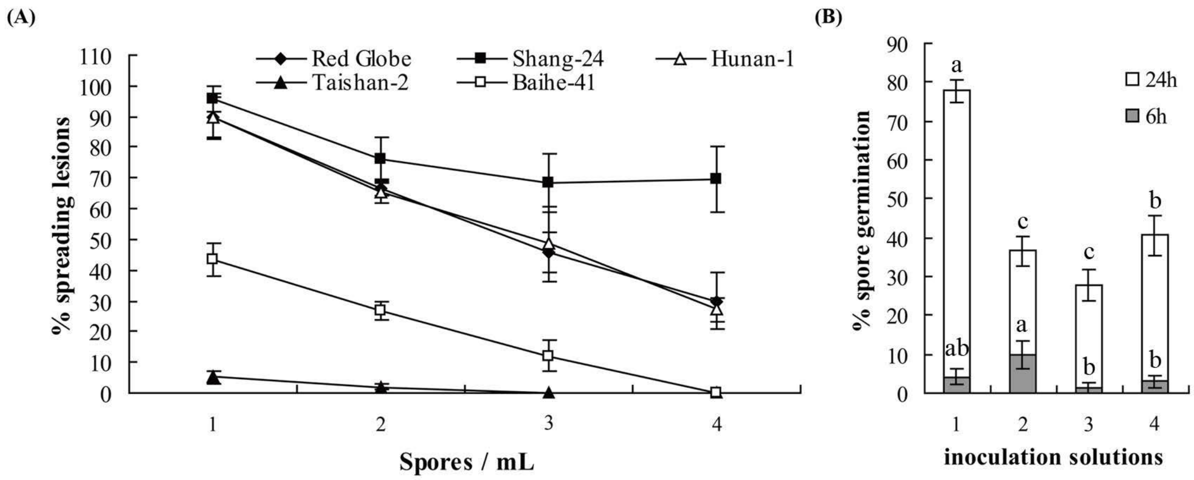

Fig. S1 Determination of the optimal inoculation solution and concentration of B. cinerea. (A) Percentages of conidia germinating at 6h (gray) and 24h (white) in solutions with different glucose (Glc) and phosphate concentrations: 1, sterile water and 1×10^6 conidia mL^{-1}; 2, 0.1 M Glc, 67 mM KH_2PO_4 and 1×10^6 conidia mL^{-1}; 3, 0.05 M Glc, 33 mM KH_2PO_4 and 1×10^6 conidia mL^{-1}; 4, 0.01 M Glc, 6.7 mM KH_2PO_4 and 1×10^6 conidia mL^{-1}. (B). Percentages of lesions spreading on detached leaves from five Vitis genotypes 4 dpi. 'Red Globe' (*V. vinifera*) and four Chinese wild Vitis 'Shang-24' (*V. quinquangularis*), 'Hunan-1' (*V. pseud oreticulata*) , 'Taishan-2' (*V. adstrica*) and 'Baihe-41' (*V. sp.* [Maihuang grape]) were evaluated after spraying different concentrations of conidia suspensions: 1, 1×10^7 conidia mL^{-1}; 2, 1.5×10^6 conidia mL^{-1}; 3, 5×10^5 conidia mL^{-1}; 4, 5×10^4 conidia mL^{-1}. Data represent the means of three experiments. Bars represent standard deviations.

Different letters indicate statistically significant differences between different treatments (Duncan's multiple range test; $P < 0.05$)
