# Supplementary material for: Resistance evaluation of Chinese wild Vitis genotypes against Botrytis cinerea and different responses of resistant and susceptible hosts to the infection
Source: Front Plant Sci. 2015 Oct 26;6:854. doi: 10.3389/fpls.2015.00854 (PMC4620147; doi:10.3389/fpls.2015.00854)
Supplement: Supplementary file 3 [file Image2.PDF]

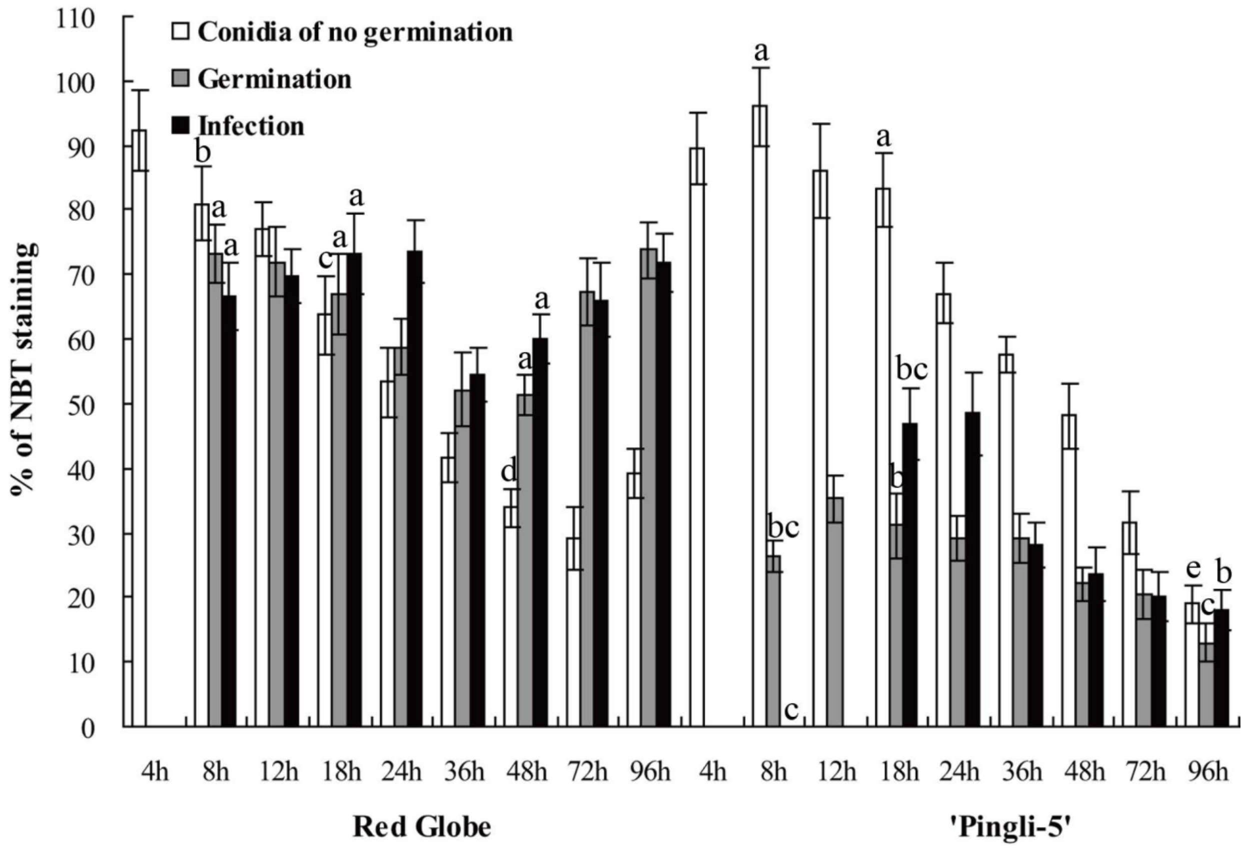

Percentages of B. cinerea conidia, grem tubes and infection sites exhibiting superoxide anion accumulation at the indicated times. At least 300 conidia were counted at each time point. These experiments were repeated three times with similar results. Bars represent standard deviations. Comparisons of statistical significance were made for the three indices. Different small alphabetical letters indicate statistically significant differences between different interactions of “Red Globe” and “Pingli-5” with B. cinerea at the indicated time points (Duncan’s multiple range test; $P < 0.05$).
